# Arginine metabolism has a pivotal function for the encystation of *Giardia duodenalis*

**DOI:** 10.1371/journal.ppat.1013851

**Published:** 2026-01-08

**Authors:** Christian Klotz, Ricarda Leisering, Kari D. Hagen, Hannah N. Starcevich, Antonia Müller, Christoph Ewald, Samuel Türken, Malte Marquardt, Saskia Schramm, Totta Ehret Kasemo, Stefanie Marek, Frank Seeber, Ralf Ignatius, Scott C. Dawson, Toni Aebischer

**Affiliations:** 1 Department of Infectious Diseases, Unit 16 Mycotic and Parasitic Agents and Mycobacteria, Robert Koch-Institute, Berlin, Germany; 2 Department of Microbiology and Molecular Genetics, University of California Davis, Davis, United States of America; 3 MVZ Labor 28, Berlin, Germany; 4 Institute of Microbiology, Infectious Diseases and Immunology, Charité-University Medicine Berlin, Campus Benjamin Franklin, Berlin, Germany; Universität Bern: Universitat Bern, SWITZERLAND

## Abstract

Arginine metabolism plays a key role in the energy metabolism of the intestinal parasite *Giardia duodenalis*, an amitochondrial protozoan that infects humans and animals and causes significant morbidity. Despite that an arginine deiminase (ADI) has been implicated in virulence*,* it remains unknown if ADI allele variants from the different genetic *G. duodenalis* subgroups (assemblages) differ in function. Here, the hypothesis was tested that sequence variation detected between *G. duodenalis* ADI alleles from the two *G. duodenalis* assemblage types found in humans affects functional parameters of the enzyme with potential consequences in life cycle progression. The ADI enzyme’s affinity for arginine was ~ 5fold reduced in sub-assemblage AII isolates, a human specific assemblage, in comparison to zoonotic sub-assemblage AI and B isolates. We identified the two amino acid residues responsible for the lower substrate affinity of ADI_AII_ variant. By combining genetic ADI-knockout mutants, biochemical assays of substrate affinity, and cellular analyses of life-cycle progression, we demonstrate that ADI is required for efficient parasite encystation and that the lower substrate affinity in ADI_AII_ correlates with reduced encystation efficiency. We further demonstrate that arginine is required for efficient encystation, and use an ADI knockout strain to confirm that ADI mediates this arginine dependence. Thus, we suggest that ADI is a quantitative trait that affects life cycle progression of *G. duodenalis* with putative clinical and epidemiological relevance.

## Author summary

In the human pathogenic parasite *Giardia duodenalis*, arginine deiminase (ADI) mediates the first step in the arginine dihydrolase pathway (ADH), metabolizing arginine to provide chemical energy in form of ATP. The bacterial-derived ADH pathway had been inherited by horizontal gene transfer, and ADI has been proposed as a virulence factor. Combining biochemical and genetic approaches with ADI knock-out mutants, we show here that arginine and its metabolizing enzyme ADI are essential for efficient life cycle progression (encystation) to form infectious cysts. Furthermore, we show a drastically impaired arginine substrate affinity for the human-specific *G. duodenalis* genotype AII in comparison to the zoonotic genotypes AI and B and identified the molecular entities responsible for this altered substrate affinity. This lower substrate affinity also correlated with lower cyst formation *in vitro* by the AII genotype.

## Introduction

*Giardia duodenalis* (syn. *G. lamblia*, *G. intestinalis*) is a medically important protozoan parasite infecting the small intestine of mammalian hosts. *Giardia* has a biphasic life cycle consisting of the motile, replicative trophozoite stage that colonizes and proliferates in the small intestine which differentiates into an environmentally resistant cyst stage. Transmission occurs via ingestion of cysts, which are formed through a tightly regulated differentiation process known as encystation. This developmental transition is essential for parasite survival outside the host and for initiating new infections, making encystation a critical step in the transmission cycle.

Advances in genotyping and its application to epidemiological studies revealed a complex population structure in *G. duodenalis* [1,2]. According to genetic information, these parasites can be grouped into eight distinct assemblages (A-H) [1]. Of these, assemblages A and B are found in humans and a broad range of domestic and wildlife animals [1]. The remaining assemblages show more narrow host ranges, with assemblage C and D found in canids, E in hoofed animals, F in felids and H in mammalian sea life [1].

Infections with different assemblages have been correlated with differences in clinical symptoms [3], and although this remains a matter of debate, this could imply that differences in virulence between parasites are genetically encoded. Interestingly, the proportions of sporadic assemblage A and assemblage B infections in humans - which are not associated with outbreaks - are uneven, with assemblage B being overall more prevalent [3–6]. Recently, improved genetic typing schemes revealed that human assemblage A infections are predominantly caused by sub-assemblage AII, a sub-assemblage type only found in humans, and only rarely by assemblage AI, a sub-assemblage of animals and humans [6–8].

The parasite-derived factors and molecular mechanisms that cause clinical disease, modulate virulence, and determine differences in parasite assemblage distribution in humans are still largely undefined [9–12]. Nonetheless, several molecular virulence factors have been proposed [9,10]. Arginine deiminase (ADI) is one putative virulence factor that is proposed to provide multiple virulence-associated functions in the parasite and its interaction with the host [13–15]. For example, *G. duodenalis* ADI has been shown to potentially interfere with NO-dependent anti-parasite defense [16,17] and also with epithelial cell proliferation through arginine consumption [18]. Competition by parasites for arginine with the host epithelium or microbiome thus may induce host pathobiology and immune modulation [16,17].

**Fig 1:**
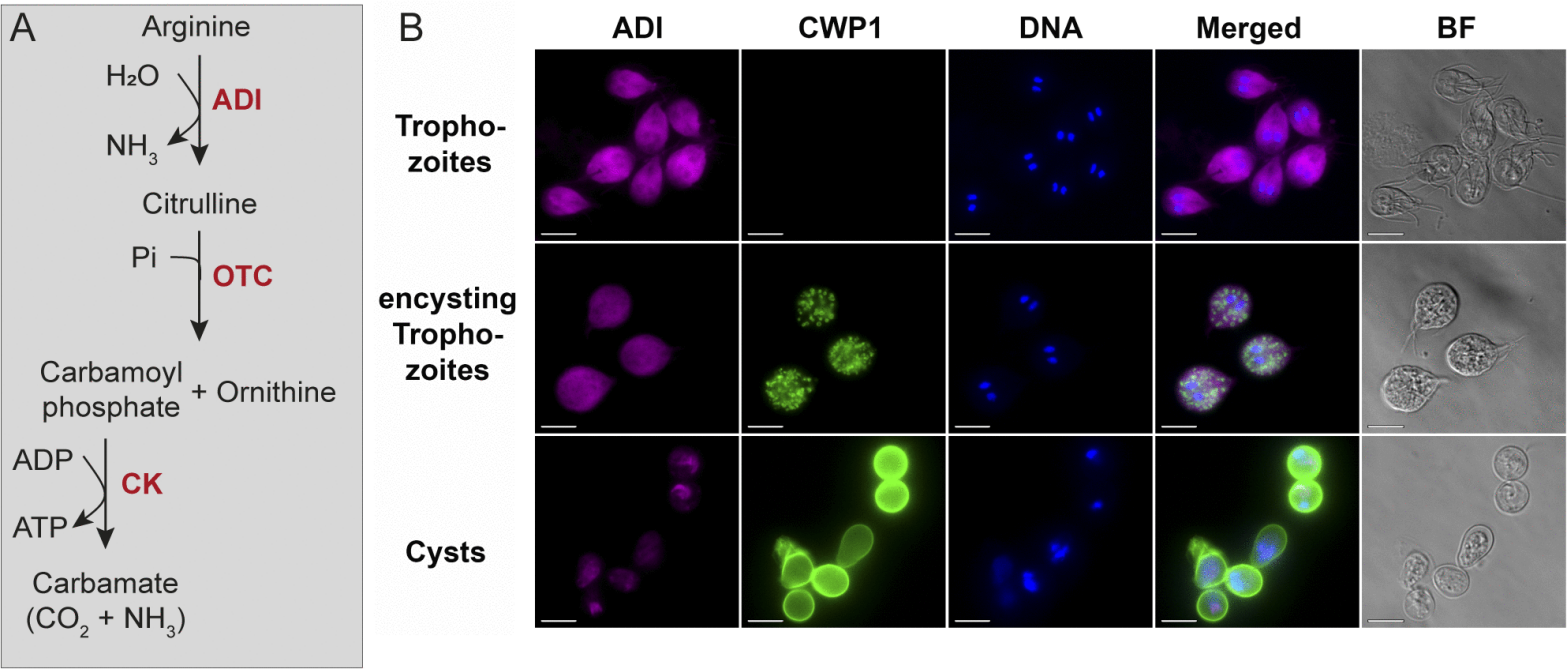
Schematic of the arginine dihydrolase (ADH) pathway with localization of arginine deiminase (ADI) in different life cycle stages of *G. duodenalis.* **(A)** Overview of the ATP-generating ADH pathway, including the key enzymes in red: arginine deiminase (ADI), ornithine-transcarbamylase (OTC) and carbamate kinase (CK). **(B)** Cytoplasmatic localization of ADI with custom antibody in trophozoites and *in vitro* encysting trophozoites and cysts (the latter both 24 h after induction of encystation). Bright field (BF) and immunofluorescence images of WB clone C6 parasites are shown using a polyclonal alpaca antiserum raised against recombinant ADI protein (magenta), anti CWP1-antibody (Giardi-a-glo, green), and nucleic acid stain Hoechst (blue). Images were acquired with equal exposure time and scaling. Scale bar = 10 µm.

The ADI enzyme in *G. duodenalis* mediates the first catalytic step of the arginine dihydrolase (ADH) pathway (Fig 1A) and catalyzes the conversion of arginine to citrulline releasing NH_3_ [19]. Notably, ADI of *G. duodenalis* was an early example of a trans-kingdom horizontal gene transfer from bacteria to eukaryotes [19].

As an amitochondrial parasite lacking respiratory metabolism, substrate level phosphorylation by the glycolytic, pyruvate, and the ADH pathways provides ATP and reducing equivalents for metabolism. While ATP generation by any of the three pathways may contribute to different aspects in *Giardia* biology, including life cycle transitions or metabolic interactions with the host, energy generation may also be limited or regulated by local substrate concentrations of glucose or arginine or local oxygen concentrations. Despite the lower energy yield per substrate molecule *Giardia* trophozoites prioritize ATP generation from arginine using the ADH pathway consisting of three enzymes (one net ATP, see Fig 1A) over energy production from glycolysis (two net ATPs). Enzymes of the ADH pathway are amongst the most highly expressed metabolic enzymes in trophozoites [20–23], which furthermore underscores their importance in *Giardia* biology.

We have previously shown that *in vitro* depletion of arginine by *G. duodenalis* ADI modulates cytokine production and surface marker expression of activated human monocyte-derived dendritic cells, with potential negative consequences for adaptive immune responses [24]. Thus, *G. duodenalis* ADI may influence parasite virulence, pathogenesis, and host immunity by several modes of action. *G. duodenalis* ADI has also been proposed to have a role during encystation through histone modification [15]. Yet, ADI’s contribution to life cycle progression and, by extension, transmission has not been further examined.

Comparison of ADI sequence information in genomes of the human relevant *G. duodenalis* (sub)-assemblages A and B [25–29] indicates various grades of sequence polymorphisms, in particular between assemblage A and B whose genomes only share approximately 70% sequence homology [27,28,30]. ADI is a single gene that contains only one exon encoding a protein of 580 amino acids. The functional relevance of ADI gene sequence polymorphisms remains unexplored, despite their likely importance in mediating varied impacts of ADI in parasite biology.

Here, we show that two of three specific amino acid polymorphisms that distinguish ADI from assemblage AII from that in AI and B parasites are responsible for changes in the *K*_m_ and catalytic efficiency of the enzyme. The AII strain alleles lead to lower substrate affinity of ADI_AII_. Using knock-out experiments we further show that ADI activity is a major factor boosting encystation rates. This work provides the first evidence that allelic variation in the ADI enzyme constitutes a functional polymorphism that can directly impact encystation efficiency in *G. duodenalis*. The observed differences in ADI kinetics among assemblages likely reflect evolutionary adaptations to varying arginine availability in different host environments, supporting the ADI enzyme’s role as a context-dependent virulence factor.

## Results

The ADH pathway is implicated in key roles for microaerophilic *Giardia*, and as shown in Fig 1A, ADI catalyzes the first step in the ADH pathway by converting arginine into citrulline and ammonia. As seen in Fig 1B, immunofluorescence analysis revealed that ADI is localized to the cytoplasm of both metabolically active and encysting trophozoites. In the metabolically less active cyst form, however, the ADI signal was reduced and, with respect to localization, was unevenly distributed throughout the cytoplasm (Fig 1B). Both observations confirm previous reports of ADI protein abundance and localization in proliferating trophozoites and during encystation [22,31].

### ADI allelic variants from human-derived *G. duodenalis* assemblage AII isolates possess lower arginine substrate affinity than ADI from zoonotic assemblages AI and B

Cellular energy metabolism, including arginine metabolism, may vary between different *G. duodenalis* assemblages. While ADI is known to play roles in diverse aspects of *G. duodenalis* virulence and biology, such as encystation [15], the relevance of putative ADI activity variants in distinct *G. duodenalis* assemblages and their impact on aforementioned traits remains unknown and was therefore evaluated.

**Fig 2:**
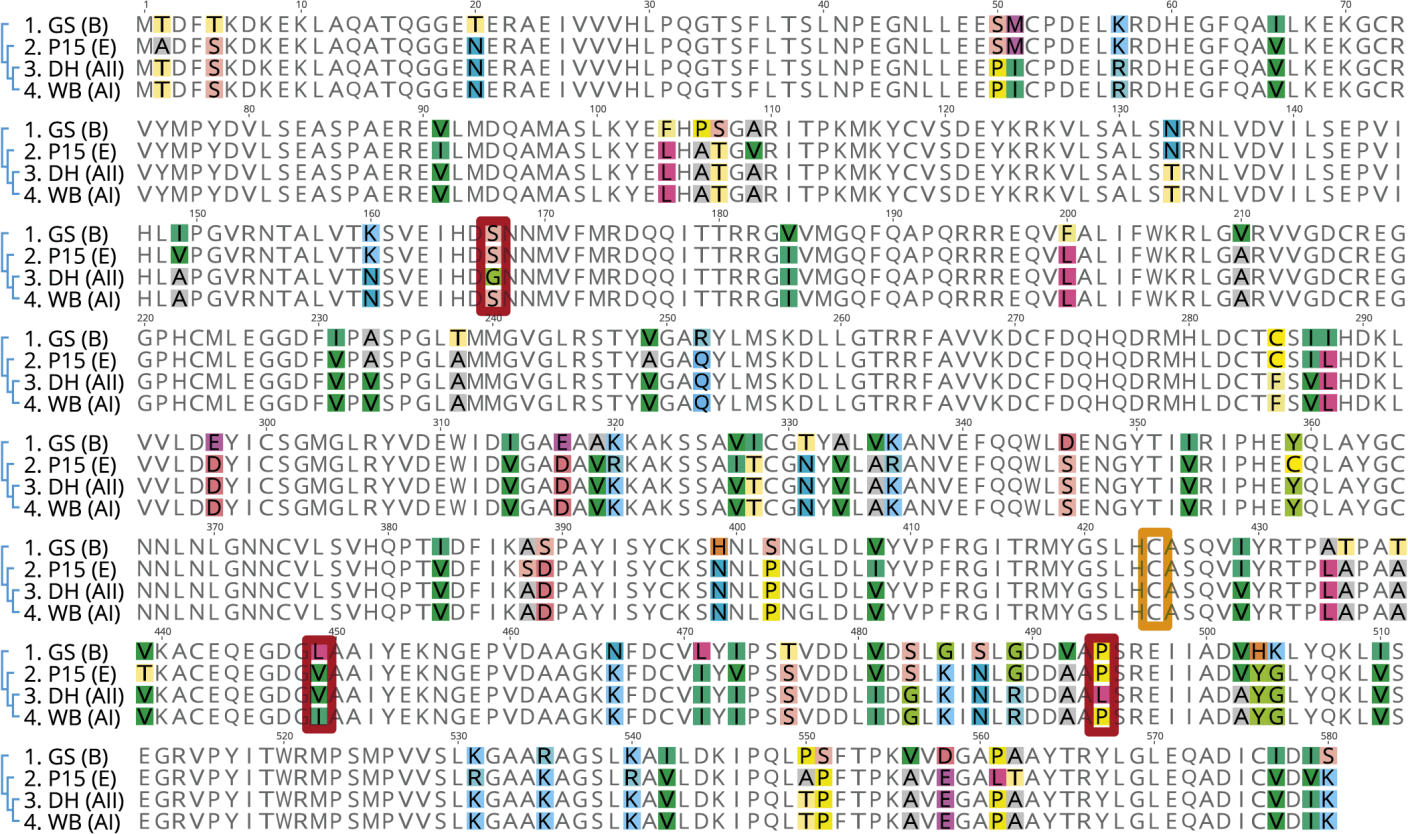
Variation in the amino acid sequences of ADI within *G. duodenalis* reference isolates. Variant amino acids of ADI from assemblages AI (isolate WB clone C6), AII (isolate DH), B (isolate GS), and E (isolate P15) were color-coded with random color selection to highlight the amino acid sequence variation. The cysteine at the active center at position 424 (boxed orange) and the three differing amino acids (boxed red) for assemblage AI (WB) and AII (DH) sequences at positions 167, 449, and 494 are marked.

**Fig 3:**
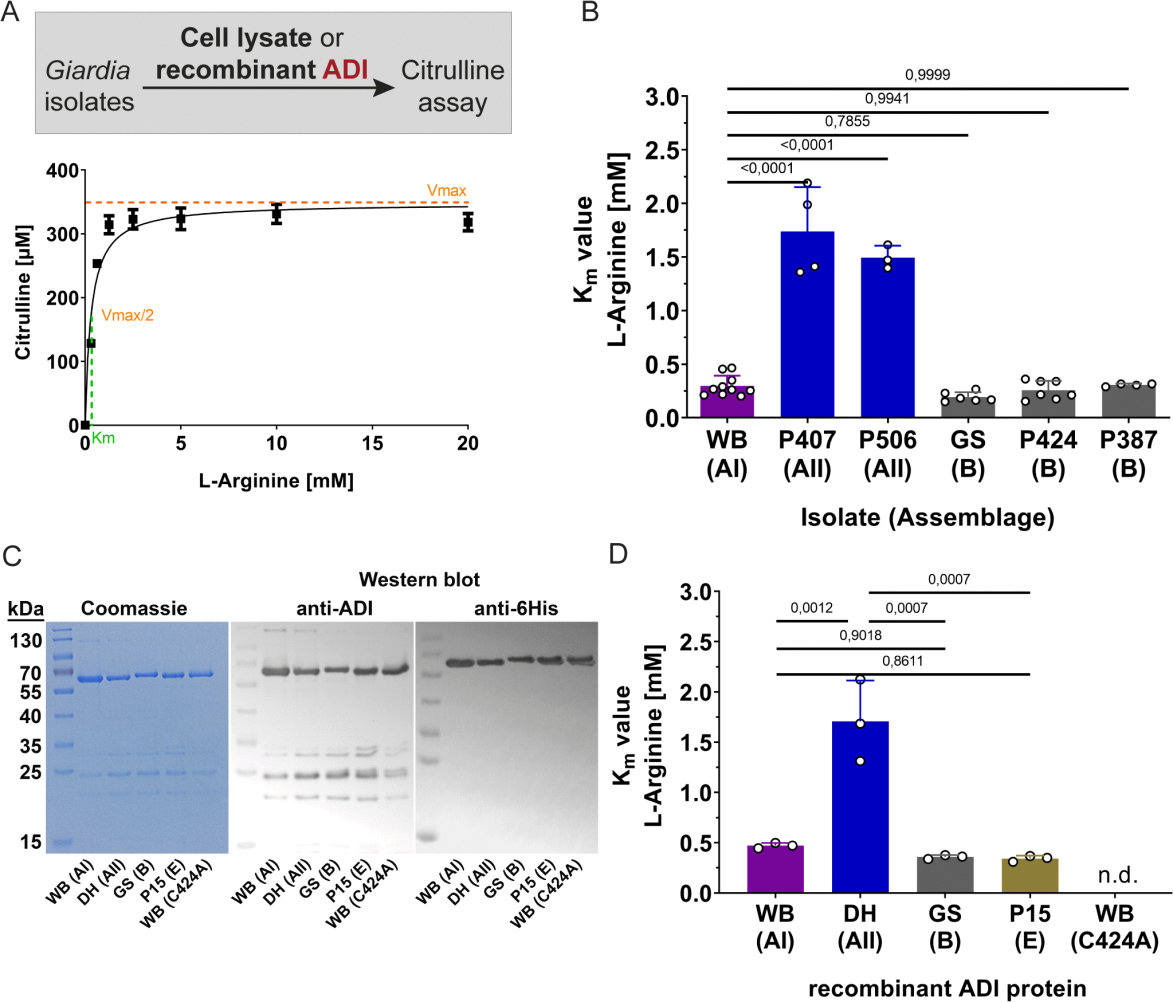
The assemblage AII ADI enzymes display a lower substrate affinity as compared to ADI in assemblages AI or B isolates. **(A)** Schematic representation and experimental example for the determination of the ADI *K*_m_ value for arginine. For determination of Michaelis-Menten kinetics for the respective *G. duodenalis* assemblage type ADI proteins, either the cell lysate or the recombinant ADI proteins were incubated with increasing concentration of the arginine substrate and formation of citrulline was quantified. **(B)** *K*_m_ values (mean ± SD) of ADI activity in lysates of *G. duodenalis* trophozoites demonstrate the significantly lower substrate affinity of ADI_AII_ enzymes from the assemblage AII isolates as compared to ADI_AI/B_ from assemblage AI and B isolates. Titration curves to determine *K*_m_ values are shown in S1 Fig. Statistical testing was done using one-way ANOVA and Tukey post hoc tests of n = 3-10 independent experiments (shown as dots) per isolate each performed in technical triplicates (exact p values against *K*_m_ value of WB (AI-type) ADI are presented). **(C)** Recombinantly expressed 6His-tagged ADI proteins representing assemblages AI (isolate WB C6), AII (isolate DH), B (isolate GS), E (isolate P15) and an inactive ADI_AI_ (C424A) were affinity purified and analyzed by SDS-PAGE with Coomassie Blue staining as well as by Western blot analysis using a polyclonal alpaca anti-ADI serum [24] or an anti-6His antibody. For qualitative assessment equal volume (mixed 3:1 with 4x SDS sample buffer) of each purified protein sample was loaded (corresponding protein amounts: WB C6, 3.15 µg; DH, 2.25 µg; GS, 2.16 µg; P15, 2.12 µg; ADI_AI_ C424A, 1.87 µg) **(D)** *K*_m_ values for each of the recombinant ADI proteins are shown. Protein sequences of ADI enzymes in lysates of assemblage AII isolates P407 and P506 in (B) are identical to the sequence of recombinant enzyme deduced from reference isolate DH. *K*_m_ values represent the mean ± SD of three independent experiments each performed in triplicates. Titration curves to determine *K*_m_ values are shown in S2 Fig. Statistical analyses shown used a one-way ANOVA and Tukey post hoc test (exact p values are presented).

To define potential functional variation of arginine metabolism in different *G. duodenalis* assemblages, we first investigated ADI sequence variation in *G. duodenalis* reference strains (Fig 2) and enzymatic activity (Fig 3A, 3B) in representative clinical *G. duodenalis* isolate cultures previously established in our laboratory. These clinical isolates included assemblage AII strains (isolates P407 and P506) and assemblage B strains (isolates P424 and P387) [28,32] and were compared to the well-studied reference strains WB clone C6 (assemblage AI) and GS (assemblage B). Together, these isolates provide examples of assemblages AI, AII and B found in humans and are all culturable axenically *in vitro*. Except for a few assemblage E isolates, no successful *in vitro* cultures of other *G. duodenalis* assemblages have been described.

In Fig 2, the sequence alignment of ADI proteins predicted from reference strains that we retrieved from the public database giardiadb.org [26] is shown. As expected, over the complete ADI gene sequence length of 580 amino acids the sequence of assemblage AII (DH isolate) only displayed three different amino acids as compared with assemblage AI (WB isolate) but differed at 34 and at 64 amino acid positions in comparison with assemblage E (P15 isolate) and B (GS isolate), respectively (Fig 2).

Strikingly, at the positions of the three amino acids that differed between assemblage AI and AII (S167G, I449V, P494L), the assemblage B and E sequences are identical at two positions (S167, P494) compared to the sequence of assemblage AI. At position 449, assemblage B and E possess leucine and valine residues, respectively, which have comparable biochemical characteristics to the cognate isoleucine at position 449 in the assemblage AI sequence (Fig 2).

To determine potential functional differences of ADI variants from different assemblages, we first generated cell lysates of *in vitro* cultured trophozoites from isolates of each of the different *G. duodenalis* assemblages and determined the substrate affinity of respective ADIs. By quantifying the product citrulline (using a standard assay), we determined the *K*_m_ values assuming Michaelis-Menten kinetics (Fig 3A). Overall, the *K*_m_ values of ADI_AII_ were approximately 5-fold higher than the *K*_m_ values from either the ADI_AI_ or ADI_B,_ which showed similar *K*_m_ values (Figs 3B; S1).

### Recombinant ADI variants confirm substrate affinity differences between *Giardia* assemblages

The different substrate affinity of ADI_AI_ and ADI_AII_ was unexpected, as the two sub-assemblages share an overall high ADI sequence homology in comparison to ADI_B_ (see Fig 2). Notably, also based on whole genome level, assemblages AI and AII share sequence homology of approximately 99% [25,28], whereas assemblages A and B only share approximately 70% sequence homology [33]. We therefore next asked whether the differences in enzymatic activity can be assigned to the three different amino acids in the underlying ADI_AI/AII_ sequences.

To confirm that the apparent *K*_m_ values were indeed due to the ADI enzymes and not due to unknown confounding activities in the parasite lysates, we recombinantly expressed ADI_AI_, ADI_AII_ and ADI_B_ in bacteria and determined the *K*_m_ values by using the different affinity-purified enzymes (Figs 3C, 3D; S2). We also included a recombinant ADI_E_ of assemblage E (P15 isolate), an assemblage type that has been occasionally described in human infections. For further comparison, an engineered inactive recombinant variant of ADI_AI_ C424A was included as well [24,34]. Overall, the *K*_m_ values of the recombinant proteins closely matched the *K*_m_ values observed in the trophozoite lysates from the respective assemblages. Again, the recombinant ADI_AII_ had a significantly decreased substrate affinity (Figs 3D; S2). Of note, the ADI_E_ showed a similar *K*_m_ value as the ADI_AI_ and ADI_B_. The inactive ADI protein expectedly showed no detectable substrate conversion (Figs 3D; S2).

### Identification of key amino acid residues defining ADI substrate affinity differences and their conservation in assemblage AI and AII

To test whether assemblage AII isolates generally carry the identified three amino acid differences as compared to other assemblages (Fig 2), we amplified and sequenced the ADI genes from 14 different assemblage AII isolates established previously as cultures from stool samples of individual giardiasis patients [28,32,35]. All ADI_AII_ sequences displayed the same three amino acid differences as the DH isolate as compared to ADI_AI_ (S3 Fig). Most of the ADI_AII_ sequences (n = 11) of the isolates studied were identical to the reference sequence of the DH isolate, but three patient isolates had additional private SNPs at different sites in the gene sequences (S3 Fig).

In a similar manner, we sequenced the ADI_B_ genes derived from cultures of assemblage B patient isolates to confirm the conservation of the three specific ADI variant amino acid residues that set apart ADI_AI/B_ from ADI_AII_. *G. duodenalis* parasites are binucleated organisms and possess a tetraploid genome, which leads to different degree of allelic sequence heterozygosity (ASH). Because assemblage B isolates often possess a high degree of ASH leading to ambiguous sequences using Sanger sequencing, we first cloned the ADI sequences from PCR products into a standard cloning vector and then analyzed 10 clones for each patient isolate. Altogether, for the 15 isolates tested, we identified 17 different alleles encoding the ADI_B_ protein (S4 Fig). In all ADI_B_ alleles, the positions of the three amino acids S167, L449 and P494 were conserved and identical to the GS reference isolate and ADI_AI_. Thus, our extensive analysis of ADI sequence variation in the various assemblages supports the existence of two specific amino acid mutations in the ADI_AII_ protein (at positions G167, L494) that are likely responsible for the functional differences in *K*_m_ (Figs 3B, 3D; S1–S5).

**Fig 4:**
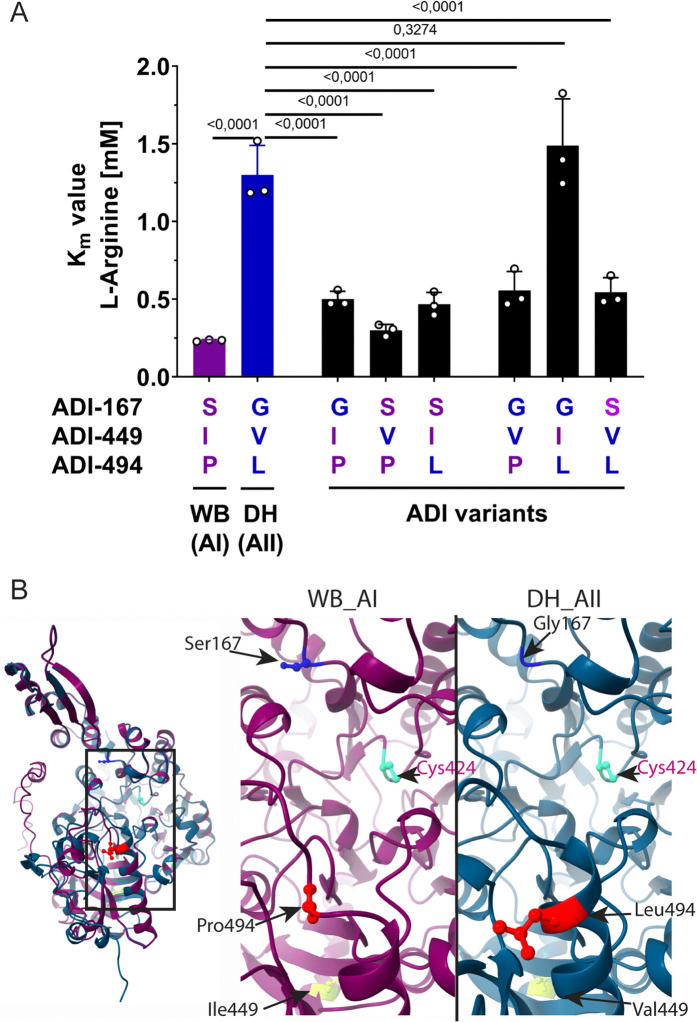
Two distinct amino acid residues near the active center confer the lower substrate affinity of ADI from the assemblage AII as compared to assemblage AI. **(A)** The arginine *K*_m_ values for recombinant ADI_AI_ variants at amino acid position 167, 449, and 494 representing either AI or AII sequence residues were compared to unmodified assemblage AI (WB) and AII (DH) isolate ADI proteins. See S6 Fig for enzymatic assays to determine *K*_m_ values. Mean ± SDs of three independent experiments each performed in triplicates are shown and analyzed by one-way ANOVA and Tukey post hoc to test for significance against *K*_m_ value of AII-type ADI (exact p values are presented). **(B)** AlphaFold models of ADI_AI_ (isolate WB C6, pink structure) and ADI_AII_ (isolate DH, blue structure). The overlay shows the overall structural similarity of both ADI variants (left). Zoom in (right) on regions encompassing the variant amino acid differences with the cysteine at the active center (pink).

To assess this hypothesis, we consecutively mutagenized the ADI encoding sequence of assemblage AI at the three differing amino acid residues (ADI-167, ADI-449, and ADI-494) to match the assemblage AII ADI amino acid sequence. The resulting ADI cDNA variants were recombinantly expressed and the *K*_m_ value determined for each variant protein. Of the eight possible variants, the double mutant ADI_AI_ (S167G and P494L) showed the functional differences in substrate affinity observed between ADI_AI_ and ADI_AII_ proteins (Figs 4A; S6). Single changes in any of the three positions had no or only marginal effects on substrate affinity. Also, the amino acid exchange I449V had no significant effect on the substrate affinity of the ADI enzyme. Notably, catalytic efficiency (k_cat_/*K*_m_) was also significantly higher in ADI_AI_ compared to ADI_AII_ (S1 Table).

Next, we retrieved the predicted protein structure for ADI of the reference strains WB (AI) and DH (AII) from AlphaFold 2 [36] and Chai-1 [37] and compared the structures using ChimeraX to reveal possible structural implications of the amino acid substitutions at ADI-167 and ADI-494 (Figs 4B, S7 Fig). Expectedly, ADI structures from assemblage AI and AII aligned well, particularly around the active center. This structural analysis, however, is consistent with the prediction that both variants, ADI-G167 and ADI-L494 as present in AII, likely may have a structural impact ipsilateral to the active center, whereas position 449 does not (Fig 4B). Further structural model predictions with arginine positioned in the active center and only considering the mutations at position 167 and 494, corroborated this idea (S7 Fig).

### An essential role for arginine metabolism in encystation in *G. duodenalis* assemblages

In the gut, completion of the *G. duodenalis* life cycle relies on successful differentiation of the trophozoite to the environmentally resistant cyst via a highly regulated developmental process [20,23,38,39]. This encystation is a highly energy-dependent process and includes incomplete division, remodeling of the endomembrane system, and the specific assembly of the cyst wall.

**Fig 5:**
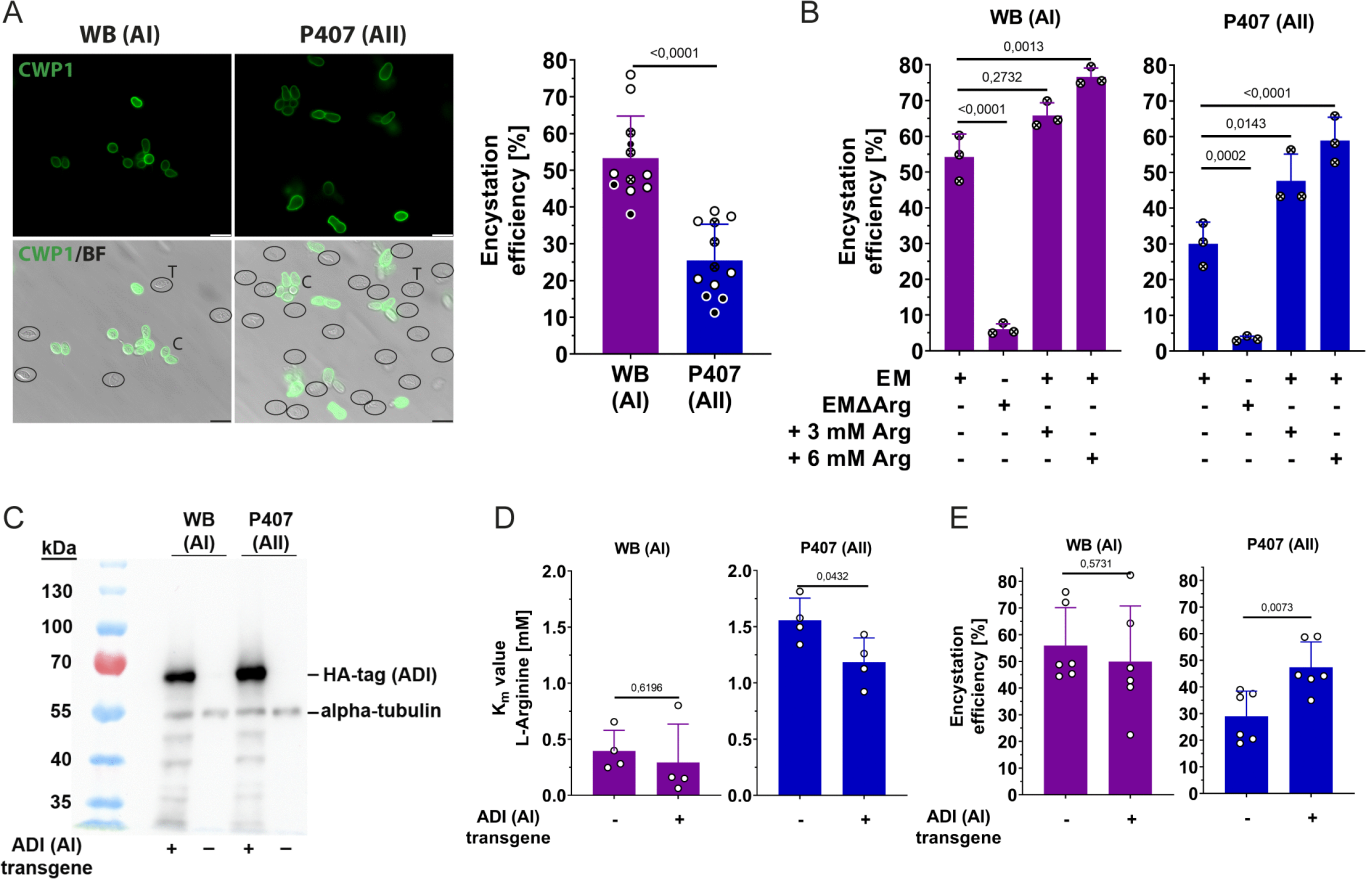
Encystation efficiency depends on arginine availability and ADI affinity. **(A)** Differences in the efficiency of *in vitro* encystation is compared between the assemblage AI isolate WB and assemblage AII isolate P407. Representative bright field (BF) and immunofluorescence images using anti CWP1-antibody (green, left) illustrate the lower encystation efficiency of P407 in comparison to WB isolate (graph, right) 24 h after induction of encystation. CWP1-negative trophozoites (T) are circled for better visualization. Note, as cell membranes were not permeabilized, only cysts (C) with CWP1 protein exposed to the outside showed strong fluorescence signal. These were counted as cysts. Scale bar = 10 µm. Dots show the mean ± SD of independent biological experiments, each performed in 3-12 replicates. Statistical analysis of p values was done by unpaired t-test. Data represent total cell counts of n = 12361 (WB) and n = 15061 (P407). Results are summarized from different experimental datasets, including the data of experiments shown in (B) and (E), as depicted by the different symbols. **(B)** The *in vitro* encystation efficiency in both assemblage types AI and AII is drastically reduced in arginine-depleted encystation media (EMΔArg) and efficiency is increased by supplemented arginine. The arginine concentration in EM was calculated to be approximately 3 mM (see S9 Fig). Values plotted represent the mean ± SD of three independent experiments. Data represent total cell counts of n = 2368 (P407), n = 2895 (WB), n = 2900 (P407, EMΔArg), n = 1294 (WB, EMΔArg), n = 2206 (P407, + 3mMArg), n = 3128 (WB, + 3mMArg), n = 2545 (P407, + 6mMArg), n = 2746 (WB, + 3mMArg). **(C)** Western blot analysis was performed using an anti-HA antibody to detect recombinant HA-tagged AI-type ADI expression constructs transfected into both P407 (assemblage AII) and WB (assemblage AI) isolates. A pan alpha-tubulin antibody was used as a loading control. **(D)** The relative arginine *K*_m_ of ADI activity in lysates is shown for both transgenic WB C6 (AI) and P407 (AII) trophozoites. Mean ± SD of three independent experiments in triplicates are indicated. **(E)** Increased encystation efficiency in transgenic P407 (AII) expressing additional AI-type ADI as compared to parental controls. Mean ± SD encystation efficiencies for six independent experiments comprising six replica each are shown. Data represents total cell counts of n = 8394 (P407), n = 6278 (WB), n = 6869 (P407:ADI-AI), n = 8684 (P407:ADI-AI). Statistical analyses in (B), (D), and (E) were performed using a one-way ANOVA and Tukey post hoc test (exact p values are presented).

Building on our comparisons of ADI substrate affinities in different assemblages, we therefore hypothesized that the lower ADI substrate affinity of ADI_AII_ may also affect the overall encystation efficiency of AII isolates. Encystation efficiency is defined here as the ratio of CWP1-positive cysts produced over a fixed period (24 hours) to total cell numbers, with equal starting trophozoite numbers under identical conditions (see Fig 5A). This measure reflects the overall capacity of trophozoites to complete the differentiation process to cysts. Reduced encystation efficiency in this assay could be the result of delayed or slower doubling times, defects in cyst wall biosynthesis, energy metabolism, or developmental commitment. Thus, using a standard encystation protocol, we compared the encystation of isolates WB (AI) and P407 (AII). The mean encystation efficiency of AI trophozoites (~55%) was indeed significantly higher than that of AII trophozoites (~30%) (Fig 5A). Differences in encystation efficiency were not due to a delayed encystation of P407, as encystation was not significantly changed after 48 hours compared to 24 hours (S8 Fig).

To confirm the essential role of arginine metabolism in encystation, we first enzymatically depleted arginine from the medium using the recombinantly expressed ADI_AI_ protein and then compared the encystation of AI vs. AII isolates (Fig 5B). Encystation efficiency was dramatically decreased for both AI WB and AII P407 isolates in depleted medium, with AII P407 cells showing a residual encystation efficiency of about 5% overall (Fig 5B). In contrast, supplementation of an additional 3 or 6 mM arginine to standard encystation medium (modified TYI-S-33, [40]), which already contains approximately 3 mM arginine (S9 Fig), led to an increase in encystation to 75% for AI (WB C6) and 60% for AII (P407; Fig 5B).

To investigate the impact of the ADI_AI_ and ADI_AII_ enzyme substrate affinity in the two variants and the link of arginine metabolism to the encystation, we generated transgenic WB C6 and P407 parasites expressing an integrated HA-tagged, type ADI_AI_ gene copy under the endogenous promoter using previously described methods [41]. Both strains had stably integrated the ADI_AI_ transgene into the genome and expressed similar ratios of the endogenous ADI and HA-tagged type ADI_AI_ protein (Figs 5C; S10). The *K*_m_ values in lysates of both wild-type and transgenic WB parasites (assemblage AI) revealed similar *K*_m_ values (Fig 5D). In contrast, ADI activity in AII (P407) parasites expressing the type ADI_AI_ enzyme exhibited a lower *K*_m_ value. This indicates that the expressed type ADI_AI_ transgene improved the resulting apparent affinity of the ADI activity in the AII (P407) transgenic parasites, which also expressed their endogenous ADI_AII_ type protein (Fig 5D). In line with this result, the transgenic AII strain encysted significantly more efficiently compared to the parental P407 strain, whereas encystation rates of the AI strains were not altered by transgene expression (Fig 5E).

For assemblage B isolates, no *in vitro* encystation protocol is available. Thus, to test the generality of the arginine dependency for encystation in different assemblages in the host, we fed mice either a diet with arginine-containing or arginine-free food pellets and infected them with assemblage B GS/H7 parasites. While both groups did not differ regarding trophozoite loads in the duodenum at day 7 post-infection, mice fed the arginine-depleted diet shed significantly fewer cysts in their feces (S11 Fig). Thus, studies both *in vitro* and *in vivo* support that arginine availability as well as ADI enzyme activity are indeed key factors for *G. duodenalis* cyst formation.

### ADI gene disruption confirms the essential role of arginine metabolism for encystation

**Fig 6:**
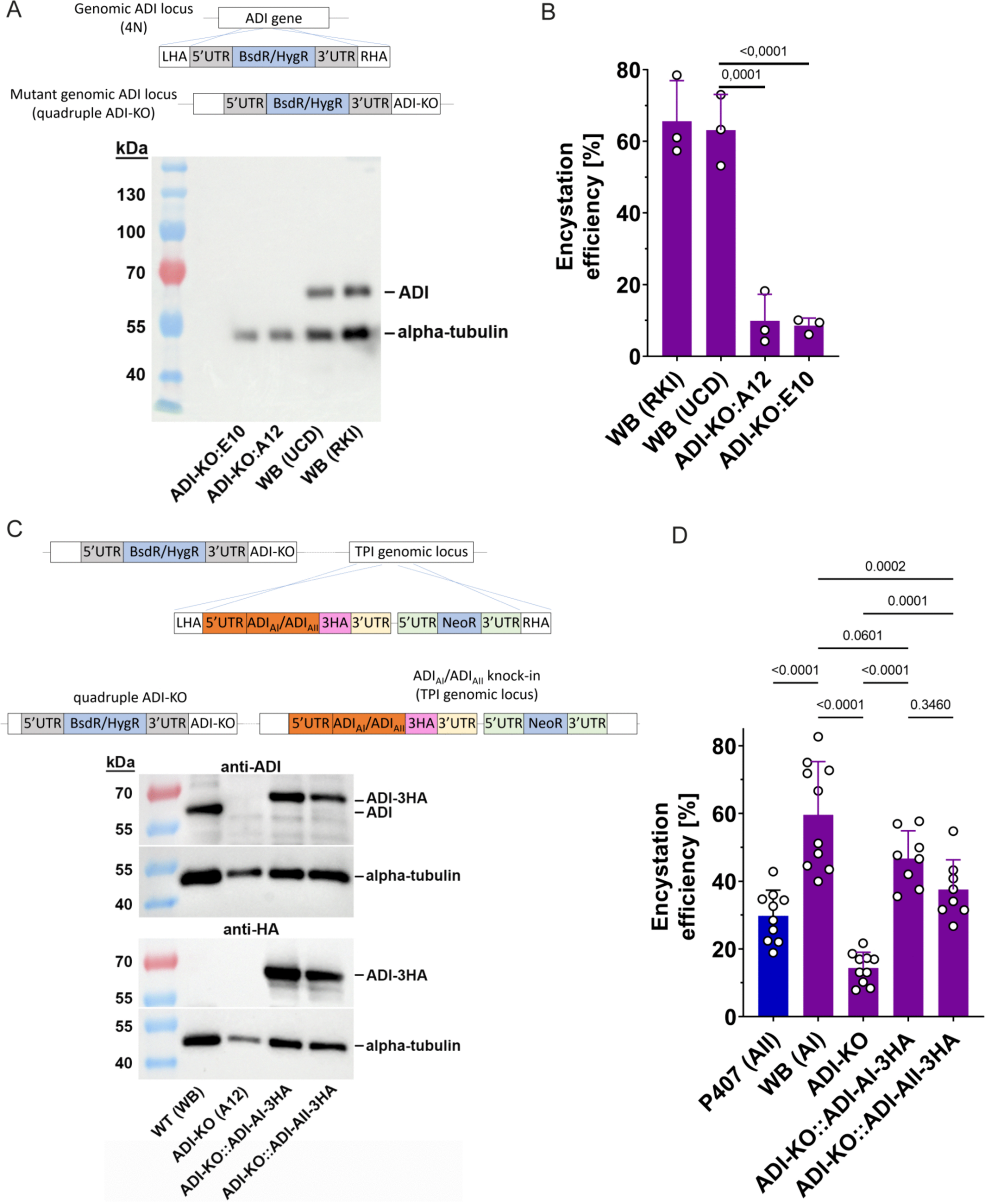
Arginine deiminase activity essential for efficient encystation in *G. duodenalis.* Quadruple allele ADI knockouts were generated using a CRISPR/Cas9 approach (see Methods and [42,43]). **(A)** The lack of ADI expression from two independent ADI knock-out clones (E10, A12) in the assemblage AI (WB) isolate was verified by Western blot analysis using a polyclonal anti-ADI antibody and a pan alpha-tubulin antibody as loading control. The parental WB (UCD) strain as well as another WB strain (RKI) maintained in a different laboratory were included as ADI-expressing controls. **(B)** The encystation efficiency (see Fig 5A) was drastically reduced in both ADI-KO clones (mean ± SD of three independent experiments, each performed in six replicates). Data represents total cell counts of n = 2450 (WB RKI); n = 2010 (WB UCD); n = 980 (ADI-KO:A12); n = 1219 (ADI-KO:E10). **(C)** Genetic complementation of ADI-KO (clone A12) was done by integrating constructs, containing either 3HA-tagged ADI_AI_ or ADI_AII_ under control of its endogenous promoter, into a separate genomic locus (TPI gene locus). The ADI expression from the two complemented ADI-KO strains was verified by Western blot analysis using a polyclonal anti-ADI and anti-HA antibodies. A pan alpha-tubulin antibody was used as loading control. The parental WB strain as well as ADI-KO (A12) were included as controls. **(D)** The drastically reduced encystation efficiency (see Fig 6B) of the ADI-KO was significantly reverted in both complemented strains. Mean ± SD of 8-10 independent experiments performed in 3-6 replica are presented. Data represents total cell counts of n = 3552 (P407), n = 7287 (WB), n = 4422 (ADI-KO), n = 6454 (ADI-KO::ADI-AI-3HA), n = 7606 (ADI-KO::ADI-AI-3HA). Statistical tests in (B) and (D) were performed by using one-way ANOVA and Tukey post hoc test (exact p values are shown). Note, P407 has a different genetic background and was therefore only tested against WB C6 for statistical significance, not against genetically modified strains with WB C6 background.

Finally, we used a new genetic strategy for *G. duodenalis* [42] to create an ADI knockout mutant to prove that a functional ADI enzyme is essential for efficient encystation in *G. duodenalis.* Using CRISPR/Cas9 we created an ADI knockout mutant (ADI-KO) strain by consecutively disrupting all four ADI alleles in the reference strain WB using two antibiotic resistance markers [42]. The absence of ADI protein expression in ADI-KO strains was confirmed in clones by both, Western blot (Fig 6A) and the lack of ADI activity in ADI-KO cell lysates (S12 Fig). While two ADI-KO clones showed sustainable growth in standard growth medium, each had decreased generation times compared to wild type parasites (~10 hours for ADI-KO versus ~7 hours for wild-type). Strikingly, cyst production of ADI-KO parasites in standard encystation medium was drastically reduced in comparison to wild-type parasites (Fig 6B). To confirm that this effect is not due to off-target effects, we performed add-back experiments and integrated ADI gene copies of HA-tagged ADI_AI_ and ADI_AII_ into the ADI-KO mutant at a different genomic locus (TPI gene locus). The complementation was confirmed by PCR approaches as well as sequencing of integrated ADI versions (S13 Fig). Western blot experiments also confirmed the expression of HA-tagged transgenes (Fig 6C). Both complemented parasite strains showed a significant reversion of the encystation phenotype from the ADI-KO (Fig 6D), indicating indeed that functional ADI is essential for efficient encystation in *Giardia*. The encystation efficiencies of the add-back ADI_AI_ and ADI_AII_ were not significantly different. This is in contrast to the encystation efficiencies of assemblage AI (WB) and AII (P407) parasites in Fig 5A. As the ADI-KO was generated in AI (WB) genetic background, we conclude that besides substrate affinity of ADI also other factors in the context of the genetic background are important to coordinate efficient encystation in different *G. duodenalis* assemblages.

## Discussion

*G. duodenalis* has a two-stage life cycle, in which massively proliferating trophozoites populating the small intestine differentiate into cysts, the transmission stage excreted with feces. Virulence, i.e., the capability of this purely luminal gastrointestinal pathogen to cause disease, is linked to parasite abundance. The association was shown by the infection dose-dependent breakdown of barrier functions in our recently developed human duodenal organoid-derived model of infection [11]. Furthermore, epidemiological data link symptomatic giardiasis disease to positive tests using less sensitive diagnostic methods [3].

**Fig 7:**
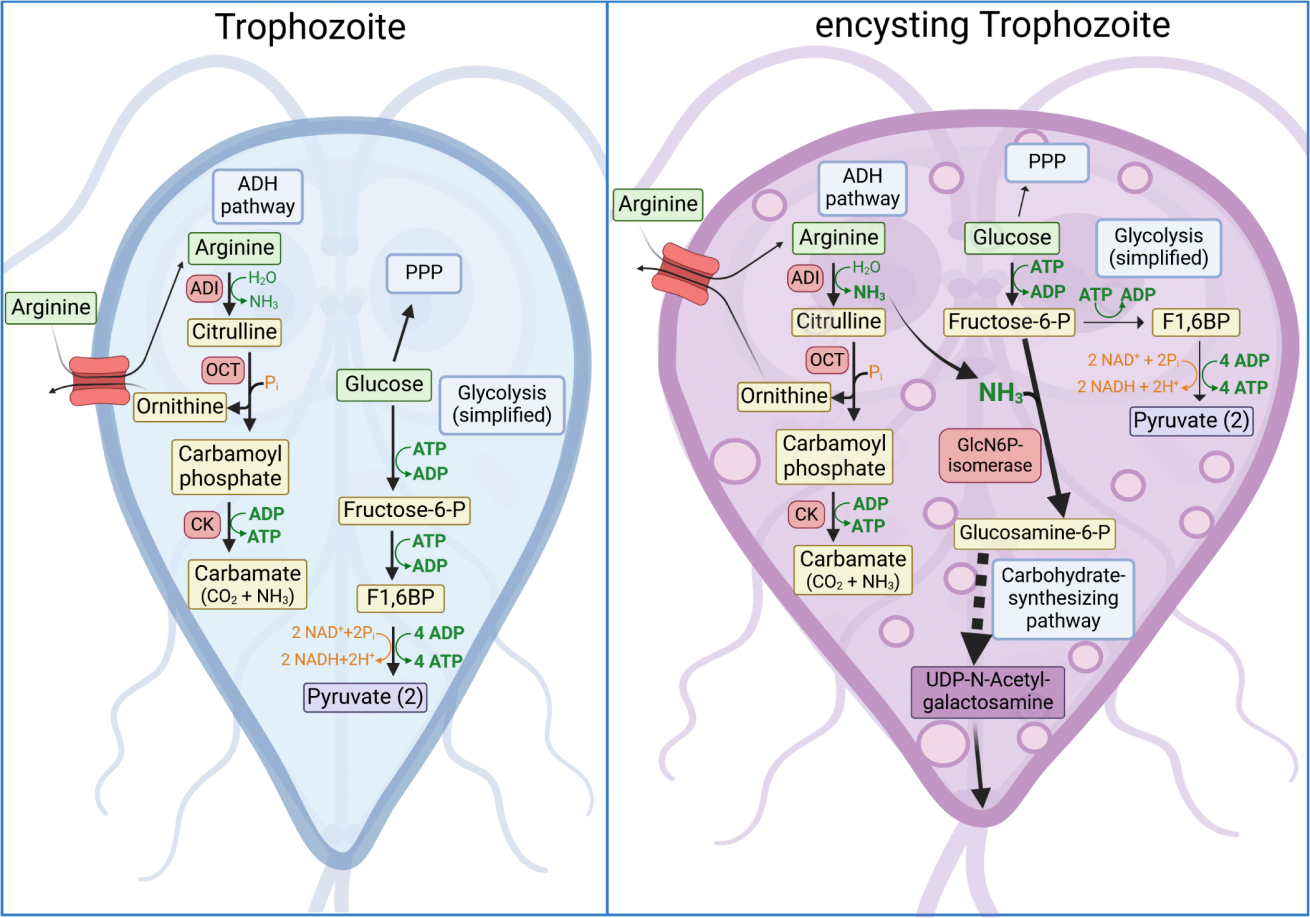
Cartoon of selected, simplified biochemical pathways for arginine and glucose in trophozoites and encysting trophozoites. In the trophozoite stage, the ADH pathway and glycolysis both represent important, redundant pathways for ATP production in this rapid growing stage. Glucose is also an important reactant for alternative pathways, such as the pentose phosphate pathway (PPP), to generate pentoses. In the encysting trophozoite, glucose is required for generation of the major carbohydrate cyst wall component UDP-N-acetylgalactosamine via a carbohydrate synthesizing pathway [43]. Other glucose-dependent pathways, such as glycolysis or PPP, are therefore downregulated. In this scenario, the ADH pathway is fundamental for energy (ATP) production. In addition, glucosamine 6-phosphate isomerase (GlcN6P-isomerase), which is the first enzyme in the carbohydrate synthesizing pathway, requires ammonium (NH_3_) to generate glucosamine-6-phosphate from fructose-6-phosphate. Created in BioRender. Klotz, C. (2025) https://BioRender.com/i19y173.

Throughout the life cycle of these microaerophilic parasites, energy in the form of ATP is derived from several substrate level phosphorylation pathways. In *Giardia*, as in eukaryotes in general, glycolysis and pyruvate metabolism yield two ATP for every glucose metabolized. In addition, *Giardia* parasites possess the ADH pathway [44,45] that can generate one ATP for every catabolized arginine substrate molecule (Fig 7). Transcriptomic and proteomic studies indicate stage-dependent metabolic adaptations that include regulation of those energy-producing pathways [20–23]. Biochemical data support the concept that ATP generation by the ADH pathway is particularly relevant for life cycle phases during which funneling of glucose into anabolic pathways in addition to catabolic pathways is required. One example is the growth-promoting role of arginine during phases of elevated biomass production, such as rapid trophozoite proliferation, when glucose may be diverted toward the pentose phosphate pathway [44]. Another example, as shown in the present study, is the contribution of the ADH pathway to encystation, where both arginine levels and ADI promote cyst formation at a stage when glucose is essential for the biosynthesis of N-acetylgalactosamine-containing cyst wall components [46]. As the first enzyme of the ADH pathway, the key role of ADI in the encystation process and the relevance of allelic forms as shown here (see also below) now firmly consolidates the proposition that ADI indeed is a quantitative trait linked to pathogenicity and transmissibility. It is intriguing that the enzymes of the ADH pathway, i.e., ADI, OTC, and CK, were likely acquired by *Giardia* from bacteria by horizontal gene transfer [19]. Conceptually, this may be considered akin to the acquisition of pathogenicity islands by bacterial pathogens [47].

### The amino acid arginine is a key component of energy metabolism in Giardia

Arginine metabolism is a key component of parasite energy production during both life cycle stages, and it is also proposed to compete for arginine with host epithelium [15,17,18,44]. ATP production by arginine metabolism is preferred over glycolysis in trophozoites, and arginine is depleted in *Giardia* growth medium during *in vitro* culture [44]. During encystation, arginine metabolism has been proposed to act as an alternative ATP source, as intermediates derived from glycolysis are potentially required for production of cyst wall components [46]. In support, genes of the ADH pathway are among the genes highly expressed in the trophozoite stage and this high expression level remains largely unchanged during encystation. Only in the later cyst stages, transcripts and protein abundances are lower [20–23]. This was also supported by our immunofluorescence analysis in Fig 1B.

Parasite arginine metabolism is also proposed to limit or modulate host immune responses in giardiasis. In the anoxic gut, competition for arginine with cells of the host epithelium [16–18] or microbiome [48] may induce both host pathophysiology and immune modulation [10]. For example, *in vitro* models with cell lines have shown that arginine depletion by the parasite hampers cell growth and NO production in intestinal epithelial cells, which may represent an immunomodulatory mechanism to dampen host immune response [16–18]. It has been speculated that this arginine depletion is mediated by extracellular ADI and other components of the ADH pathways that are increasingly released upon host cell contact [49]. In this context, it is worthwhile to mention that we observed a drastic effect on parasite encystation by media in which arginine was depleted. The depletion was acquired by addition of recombinant “extracellular” ADI suggesting that the release of ADI comes with a cost for the parasite. *Giardia* infections are characterized by Th17 T cell responses to mediate protective effects against the parasite [50–52]. The proper function of T cells depends on arginine supply [53] and it has not yet been ruled out how modulation of arginine level in the gut potentially affects anti-*Giardia* T cell responses. Thus, competition for arginine between host and parasite during trophozoite growth and/or encystation mutually contribute to host pathophysiology and/or parasite growth and transmission. With the availability of genetic tools to generate mutants in the ADH pathway as shown here combined with new *in vitro* models such as stem cell-derived intestinal organoids [11,12,43,54], these long predicted roles of arginine metabolism in *Giardia* infection can now be experimentally tested in relevant host–pathogen systems.

### The ADI pathway has both definitive catabolic and anabolic metabolic functions during *Giardia* encystation

The ADI pathway is known to play an important catabolic role in microaerophilic *Giardia* trophozoite metabolism. Here, using both biochemical and new genetic approaches in *Giardia* research, we demonstrate that a functional ADI enzyme is also essential for efficient encystation, thereby highlighting how this energy generating pathway is required for transmission of this ubiquitous pathogen. Both, genetic disruption of all four ADI alleles or depletion of the ADI substrate arginine in the culture medium led to a drastic inhibition of cyst production (Figs 5, 6). Disruption of the ADH pathway is not lethal for proliferating or encysting trophozoites, the trophozoite growth rate is nonetheless reduced. Thus, while *Giardia* uses redundant mechanisms for energy production by substrate level phosphorylation in the trophozoite stage, trophozoites require the ADH pathway to fuel the encystation process efficiently. Previous attempts to knock down ADI using siRNA-based approaches were unsuccessful [34], leading to the interpretation that ADI is essential and that genetic depletion would be lethal. In contrast, we show here that *Giardia* trophozoites lacking ADI (ADI-KO) are viable, although they exhibit a modest growth defect with slower doubling times. This demonstrates that while ADI is important for optimal trophozoite proliferation, it is not essential under *in vitro* conditions. However, its absence significantly reduces encystation efficiency, highlighting a critical role for ADI in life cycle progression and transmission. Based on results with transgenic episomally encoded overexpression a previous study [15] reported that ADI suppresses CWP-2 protein expression and cyst formation. These effects clearly differ from our finding that ADI is required for efficient encystation and rescues this property in complemented ADI-KO parasites. The data are difficult to reconcile but may be a consequence of using different clones of WB (ATCC-50582 WB clone 1267 in [15]), a different encystation protocol and different polyclonal immunoreagents to detect ADI. Reportedly, WB clone 1267 did exhibit a very low encystation efficiency of 4–8% that was reduced to 0.1% in the transgenic line derived by electroporation [15]. Unfortunately, causality between ADI overexpression in this line and decreased encystation efficiency was not assessed for example by simple cure of the transgene by relieving the selection pressure imposed via the puromycin resistance marker. We posit that a positive role for ADI in encystation is in better agreement with the results that imply a beneficial effect of arginine for WB clone C6 but also other isolates as shown here for isolate P407 of assemblage AII. Additional functions of ADI reported in [15], including interactions with VSPs and roles in antigenic variation or sumoylation, were not investigated here and remain unverified. Importantly, with the development of genetic tools enabling precise ADI knockouts, future studies can now rigorously dissect the distinct consequences of ADI overexpression versus loss-of-function and directly test whether phenotypes arise from metabolic perturbations or potential non-canonical roles such as transcriptional or epigenetic regulation.

Microbial intermediary metabolism is defined by glycolytic intermediates or other metabolic intermediates from catabolic metabolism being redirected into anabolic central carbon metabolism to support the biosynthesis of nucleotides, amino acids and lipids. *Giardia* parasites are known to use glycolytic intermediates for the production of UDP-N-acetylgalactosamine, which is an important precursor for cyst wall components [46]. Encystation may deplete these metabolic intermediates and limit energy production by glycolysis during encystation. This metabolic flexibility of intermediary metabolism allows the parasite to couple nutrient availability to life cycle progression, particularly during phases of rapid proliferation and even to cyst wall biosynthesis in encystation.

Here we suggest that intermediate products of the ADH pathway may also play an important biosynthetic role in anabolic metabolism during encystation. To synthesize UDP-N-acetylgalactosamine from endogenous glucose, *Giardia* needs to generate glucosamine-6 phosphate from fructose-6 phosphate using glucosamine-6 phosphate isomerase. It is a cytosolic enzyme with aminase activity; induced in encysting parasites and is also dependent on NH_3_ as a further substrate [46] (see Fig 7). Thus, we postulate that ADI not only provides the citrulline-intermediate for the ADH pathway-dependent ATP generation but also increases the ammonia/ammonium concentration within the encysting trophozoite. Thereby, anabolic and catabolic functions of the ADH pathway fuel glucosamine-6 phosphate synthesis and eventually cyst wall formation. ADI, OCT and CK are progressively less abundant in maturing cysts, as also confirmed for ADI by our immunofluorescence analysis (Fig 1B). Furthermore, the progressive deposition of the cell wall is thought to act as a diffusion barrier [55] and may limit arginine influx and thus act as a natural negative feedback loop to signal that cyst wall generation is completed. This could explain the modulation of encystation by the substrate affinity of ADI.

In support of this role of ADH pathway components in encystation-specific metabolism a number of groups have produced valuable inventories of transcripts and proteins for studying encysting WB parasites [20–23]. The most comprehensive study to date [22] confirms in great detail a higher abundance of enzymes required to synthesize the N-acetylgalactosamine. These components remain at a high level in encysting cells and cysts while glycolytic proteins, in particular those associated with ATP production, are downregulated.

### Variant arginine substrate affinity of ADI may impact encystation efficiency and host metabolic interactions in different *G. duodenalis* assemblages

This work provides the first direct functional evidence for genetically determined virulence in *G. duodenalis*. Moreover, we show that arginine substrate affinity of the ADI enzyme varies between different *Giardia* assemblages. The sharp distinction in ADI substrate affinity between *G. duodenalis* assemblage AI and AII was unexpected, as ADI gene similarity reaches 99.6% with only three amino acid differences on protein level between the two groups. Two conserved amino acid residues in the ADI_AII_ sequence are solely responsible for the functional difference. The structure prediction showed the positioning of the two functional mutations nearby the active center, but not in direct vicinity. In a recent study, Li et al mutated ADI from *Enterobacter faecalis* and showed similar modulation of catalytic activity in mutations affecting regions nearby the arginine binding sites [56]. With the development of new tools for genetic manipulation in *Giardia*, future work combining structural analysis and comparative functional assays to study diverse ADI orthologs will help to define structural features responsible for differences in arginine substrate affinity and catalytic efficiency.

Earlier studies showed the presence of an extremely efficient arginine/ornithine antiporter on the plasma membrane of *G. duodenalis* with *K*_m_ values as low as 15 µM [57]. There is no genetic or other experimental evidence for efficient arginine biosynthesis pathways in *G. duodenalis* [58] and efficient arginine uptake would thus be essential. For comparison, the most efficient arginine transporter (CAT-1) in human cells has a *K*_m_ value of 100–160 µM [59] -- approximately 10-fold less efficient. This difference in arginine transporters between parasite and host argues for a strong competition for free arginine [15–18]. Importantly and in contrast to, e.g., *Trichomonas* parasites, there is no intracellular free arginine pool detectable in *G. duodenalis*, which likely is a consequence of the highly active ADH pathway [60].

Recent molecular epidemiological studies indicate that assemblage AII represents an anthroponotic assemblage type only found in humans, whereas assemblage AI and B represent potentially zoonotic assemblage types populating humans and a wide range of animals [1,7,8]. Our analysis of allelic ADI variants of patient isolates indicates that the two amino acid residues distinguishing ADI of anthroponotic from zoonotic assemblages are conserved within the assemblage groups.

Differences in sequence and function of ADI_AII_ could therefore be a consequence of host adaptation. Host dietary requirements for arginine are highly species-specific, and while such requirements are well-studied in livestock species and companion animals, dietary requirements for arginine are not well understood for humans [61]. With respect to parasite ADI evolution, selection pressure in different hosts may act directly on parasite fitness with respect to the completion of the transmission stage (e.g., cyst). Hence, the broader zoonotic distribution of *G. duodenalis* assemblages AI and B, relative to the narrower anthroponotic restriction of AII, may in part reflect underlying metabolic differences, including assemblage-specific variations in ADI arginine affinity.

Overall, our observation that different *Giardia* assemblages encode ADI alleles with varying substrate affinities should not be interpreted as evidence of detrimental ADI function but rather treated as an example of adaptive metabolic variation in *Giardia* shaped by the ecological context. Arginine concentrations in the intestinal environment are known to fluctuate, both between host species and within a single host during infection due to factors such as diet, microbiota composition, immune responses, or host-parasite competition (reviewed in [62]). In this light, the existence of both high- and low-affinity ADI variants between different assemblages may reflect evolutionary tuning to different substrate availability regimes. For instance, a low *K*_m_ (high-affinity) enzyme is advantageous in environments where arginine is scarce, ensuring efficient catalysis even at low substrate concentrations. Conversely, a high *K*_m_ (low-affinity) enzyme may be better suited for high-arginine conditions, where it reduces the risk of product overaccumulation and allows more sensitive modulation of activity in response to changing substrate levels.

The presence of ADI alleles with different kinetic properties across *G. duodenalis* assemblages is therefore consistent with adaptation to diverse host environments and does not imply selective disadvantage. This view aligns with established biochemical principles: enzymes with higher *K*_m_ values are often deployed for regulation under saturating conditions, while lower *K*_m_ enzymes ensure maximal activity when substrate is limiting. Additional studies using *in vivo* and physiologically relevant *in vitro* models will help clarify the ecological significance of this variation in different *Giardia* assemblage-host infections.

## Materials and methods

### Ethics statement

The work on animals has been approved by the local authorities, Landesamt fuer Gesundheit und Soziales, Berlin, Germany, under license number G_0277-17.

### Trophozoite culture

Trophozoites of the *G. duodenalis* strains WB (WB-C6, ATCC 50803) and GS (GS-H7, ATCC 50581) were propagated in TYI-S-33 medium, supplemented with adult bovine serum, in 11 ml slanted screw capped culture tubes as previously described [40]. For passaging, confluent culture tubes were incubated on ice for 20–30 minutes to detach trophozoites, and a sufficient proportion of trophozoites was transferred into new tubes filled with fresh medium.

*G. duodenalis* parasites from patients were isolated from stool samples by *in vitro* excystation following the protocol of Rice and Schaefer [63] and cultured in TYI-S-33 medium as previously described [28,32,35]. Briefly, cysts were enriched from stool samples by sucrose gradient flotation, excysted *in vitro* and cultures established through limiting dilution in 96-well plates under anaerobic conditions. Growing isolates were transferred into culture tubes for routine culture.

### Parasite encystation

Encystation was induced according to a previously published protocol using modified TYI-S-33 medium, containing higher bile concentrations and an increased pH value [64]. In brief, trophozoites were grown to logarithmic phase between 60–80% confluency in culture tubes or 12-well plates with normal TYI-S-33 medium containing 0.5 mg/ml bovine bile (Sigma, B8381) at a pH of 7.0. It was ensured that parasites numbers and density were comparable at the time of induction of encystation. For encystation, normal growth medium was replaced by the same amount of warm encystation medium, i.e., TYI-S-33 medium containing 10 mg/ml bovine bile and adjusted to a pH value of 7.85, and incubated for 24 hours at 37°C, unless stated otherwise. Arginine concentration in culture media were determined using enzymatic assay with recombinant ADI and calculation of the equimolar citrulline concentration after complete enzymatic turnover (see details of the assay below and S9 Fig). The same approach was used to deplete arginine from the media in some experiments. Trophozoites that were cultured in cell culture plates were put under oxygen-restricted growth conditions using Anaerogen Oxoid jars containing appropriate reaction bags (Thermo Fisher, AnaeroGen #AN0025).

To quantify encystation, cultures were put on ice for 20 min to detach all parasites (trophozoites, encysting trophozoites and cysts) from the cell culture plastic. Parasites were collected, pelleted at 900 *g*, 4°C and fixed with 4% paraformaldehyde in PBS for 15 minutes at room temperature. After fixation, cells were washed twice and finally resuspended with PBS. Subsequently, cells were treated 30 minutes at room temperature with 1x Giardi-a-glo antibody labeled with FITC (Waterborne Inc.) to detect cysts and nucleic acid stain propidium iodide (final concentration of 5 µg/ml) for counterstaining. Finally, cells were washed with PBS and counted using counting chambers under conventional fluorescent microscope (Zeiss Axioscope). In this assay, cell membranes were not permeabilized so that only cysts with CWP1 exposed to the surface showed strong fluorescence signals. These structures were counted as cysts. Efficiency of encystation was calculated by dividing number of cysts with total cell count (cysts and trophozoite stages).

### Animal infection

Standard *in vitro* encystation protocols are not efficient for assemblage B parasites. We therefore used an established infection protocol in mice [65] to determine the role of arginine on encystation *in vivo*. In brief, 6–10 week-old C57/BL6 mice (female and male) were randomly divided into experimental groups and pretreated for seven days with antibiotics in drinking water ad libitum, containing 1.4 g/L Neomycin (Biomol, Germany), 1 g/L Ampicillin (Merck, Germany) and 1 g/L Vancomycin (Merck, Germany). At the same time, the diets of the experimental groups were exchanged towards “arginine-free” and “control diet” (EF Crystalline AA Arginine free and EF Crystalline AA control diet, ssniff GmbH, Arnsberg, Germany). Animals were orally infected by gavage with 5 x 10^6^ trophozoites derived from an *in vitro* culture of the *G. duodenalis* GS clone H7 (ATCC 50581, assemblage B). Feces were collected daily from day 4–7 post infection and cyst shedding was determined in suspended feces (100 mg feces per ml water) by immunofluorescent microscopy using Giardi-a-glo antibody (Waterborne inc). On day 7 post infection, animals were sacrificed and a 3 cm piece of the small intestine, starting at 2 cm from the pylorus, was cut out and frozen in 1 ml RNALater solution (Qiagen, Germany) at -70° C until further processing. For DNA extraction, intestinal tissue was first homogenized in a Pecellys 24 tissue homogenizer (Bertin Instruments, France) using a bead-mixture (50/50% 1 mm and 0.1 mm sharp-edged silicon carbide beads from Bio Spec Products Inc, pre-treated at 180°C for 2.5 h) and RLT buffer from Qiagen RNeasy Mini kit (Qiagen, Germany) with 1% RNase free 2-mercaptoethanol (Merck, Germany). Homogenates were centrifuged for 3 minutes at 21.500 g and supernatants were used to isolate DNA with the NucleoSpin Tissue kit (Macherey-Nagel, Germany) according to the manufacturers’ recommendations. Quantification of *G. duodenalis* parasites was done by a published Taqman qPCR protocol targeting the rRNA gene from *G. duodenalis* [66]. An internal amplification control was included to exclude potential PCR inhibition [67]. Genome equivalents were calculated from a standard curve produced from DNA of a known number of *in vitro* grown GS/H7 isolate.

### Localization of ADI by immunofluorescence analysis

Localization of ADI protein was analyzed in trophozoites, encysting trophozoites and cysts of WB isolate. Cysts were separated from encysting trophozoites 24 hours after induction of encystation by centrifugation at 650 *g* for 10 minutes at room temperature. Cysts were collected and washed once with H_2_O and stored in H_2_O at 4°C for 48 hours and then fixed with 4% paraformaldehyde for 15 minutes at room temperature and washed twice with PBS. Remaining encysting trophozoites were detached by putting the tube on ice for 20–30 minutes and then cells were pelleted for 10 minutes at 900 *g* at 4°C. Trophozoites were harvested from a routine culture in logarithmic phase as stated above. Trophozoites and encysting trophozoites were washed two times with PBS and then fixed with 4% paraformaldehyde for 15 min at room temperature. After further two washing steps with PBS, cells were stored at 4°C until usage. For immunofluorescence staining, cells were either immobilized on poly-L-lysin pretreated chamber slides (ibidi; Germany) or stained in suspension. First, cells were permeabilized with 0.25% TritonX-100 in PBS containing 0.75% glycine for 30 minutes and then blocked with 2% BSA in PBS for further 2 hours. Primary antibodies were diluted in blocking buffer and samples were incubated overnight at 4°C. Primary antibodies used: alpaca antiserum generated against recombinant ADI from WB isolate [24] in a dilution of 1:100. After 3 washing steps with blocking buffer for 10 minutes each, secondary antibodies (goat anti lama DyLight650, 1:300; Bethyl laboratories) and directly labeled antibody Giardi-a-Glo (FITC) were diluted in blocking buffer and incubated for 2 hours at room temperature. Cells were washed again 3 times for 10 minutes each. Finally, DNA was stained with Hoechst 33342 at a final concentration of 2 µg/ml for 30 minutes and cells were imaged in mounting medium (ibidi) at a Leica Mica microscope (Leica).

### Sequencing of isolate-specific *G. duodenalis* ADI alleles

The *G. duodenalis* ADI coding sequences of 14 assemblage AII isolates were amplified from genomic DNA by PCR using specific primers (5’-CTGACAAGCACTTCATTTACTG-3’ and 5’-CGGCGGGGGCCGGTGCTTTG-3’) and the Q5 PCR kit (New England Biolabs). PCR fragments were purified with the DNA Clean and Concentrator kit (ZymoResearch). Sequencing was performed using the same amplification primers and two additional ones (5’-GTCCGCAACACGGCTCTCGTTAC-3’ and 5’-CCGAGGCGCTTCCAGAAGAT-3’) to retrieve the complete ADI coding sequence.

To determine the alleles of the ADI coding sequence of 15 selected assemblage B isolates, modified primers were used to amplify their ADI sequence (5′-CACAGCGTTTAATTTACATCTTATAAG-3′ and 5′CTAGACATAAACATCTCAATTATTTG-3′). The majority of assemblage B isolates are heterozygotes and we therefore cloned the PCR products into a standard cloning vector (CloneJET; ThermoScientific) and analyzed plasmids of 10 single bacterial clones per isolate. Plasmids carrying the ADI coding sequence were sequenced as described above.

Sequence files of both strands amplified by respective forward and reverse primers were finally analyzed with tools implemented in Geneious Prime software (Biomatters Ltd.) and CLC genomic workbench (Qiagen).

### Generation of recombinant proteins

The recombinant *G. duodenalis* ADI proteins corresponding to assemblage AI type GL 50803_112103 and its enzymatically inactive mutant form were produced as described [24]. Assemblage AII, B as well as E encoding ORFs were designed based on their genomic sequence (Gene IDs DHA2_112103, GL50581_1575 and GLP15_4932; [26]) but codon optimized for expression in *E. coli* and custom ordered (GeneArt, Invitrogen). The DNA fragments were cloned into the expression vector pASG-IBA35 (StarGate cloning, IBA GmbH) according to the manufacturer’s manual to produce a recombinant protein with an N-terminal His_6_-tag. Site directed mutagenesis using Q5 polymerase (New England Biolabs) was done according to the guidelines by the manufacturer, creating ADI with modified coding sequences, thereby transforming assemblage ADI_AI_ consecutively into assemblage ADI_AII_ in the pASG35 expression vector. The following amino acid positions and combinations thereof were modified (S167G, I449V, P494L, see Figs 2; 4A). After transformation of pASG-IBA35_ADI into *E. coli* DH5αZ1 cells, the expressed His_6_-tagged ADIs were purified by affinity chromatography followed by imidazole removal and concentration in PBS as described previously [24]. Protein concentrations were determined with a BCA protein assay kit (Thermo Scientific Fisher). Purified proteins were stored in aliquots at -70°C. Purity of recombinant proteins was controlled by SDS-PAGE and Coomassie staining. For Western blot analysis, the following antibody combinations were used: a commercial monoclonal mouse anti-6His antibody (1:2000, Qiagen) together with a peroxidase conjugated goat anti-mouse antibody (1:5000, Bethyl laboratories); an alpaca antiserum from an animal immunized with recombinant assemblage ADI_AI_ (1:1000) together with a peroxidase conjugated goat anti-lama IgG (1:10000, Bethyl laboratories). For detection, the Pierce ECL Plus substrate kit (ThermoFisher Scientific) was used and signals were recorded on a conventional documentation system (Vilbert Fusion FX6).

### Determination of enzymatic activity and enzyme *K*_m_ value

ADI activity and *K*_m_ values of recombinant, purified *G. duodenalis* ADIs were determined by colorimetric determination of citrulline formation as described [24,57]. To determine the *K*_m_ value of native *G. duodenalis* ADI, lysates of trophozoites were prepared. Per strain, three cultivation flasks of confluent trophozoites were washed with PBS at room temperature and then placed for 30 min on ice. Cultures were harvested, combined and centrifuged (900 x *g*, 5 min, 4°C). After additional washing with ice-cold PBS, cell pellets were resuspended in 500 µl ice-cold PBS, trophozoites were counted in a Neubauer counting chamber and frozen at -80 °C. Subsequently, frozen trophozoites were thawed, exposed to three freeze/thaw-cycles using consecutive steps using liquid nitrogen and a 37°C water bath. Cell debris was removed by centrifugation for 20 min at 12000 x *g* at 4°C. Supernatants were collected and 10 µl lysate were directly or in appropriate dilution with 40 mM Hepes buffer (pH 7.0) used for the enzyme activity assay. To determine the *K*_m_ value, the substrate concentration was varied: 10 µl of different arginine concentrations, prepared by diluting 100 mM arginine stock solution with 40 mM Hepes buffer (pH 7.0), were applied to obtain final assay concentrations ranging from 0-20 mM. As negative control, 10 µl lysate was replaced by 40 mM Hepes buffer (pH 7.0) for each arginine concentration. *K*_m_ and V_max_ values were computed with GraphPad Prism 9 (GraphPAD Software, Inc.).

### Generation of transgenic and knock-out strains

For generation of assemblage AI and AII trophozoites carrying an additional genome integrated ADI_AI_ transgene, the ADI_AI_ gene (GL50803_112103), including its endogenous promotor, was PCR amplified from genomic DNA of *G. duodenalis* WB isolate using primer pairs (forward 5’GGGCCTAGGATGACTGACTTCTCCAAGGATA3’; reverse 5’ GGGGCATGCCTTGATATCGACGCAGATGTC3’) and Q5 PCR amplification kit (New England Biolabs). The PCR product was inserted by Gibson cloning procedures in vector pPAC-ORF16653-HA [68,69] replacing ORF16653 with the ADI_AI_ gene, resulting in vector pPAC-ADI_AI_-HA. The vector was linearized using SwaI restriction enzyme and trophozoites of isolates WB (assemblage AI) and P407 (assemblage AII), respectively, were electroporated (GenePulser XCell, Bio-Rad) at 800 Ω, 350 V und 960 μF with ~15µg linearized vector DNA. Transgenic parasites were selected in media containing 40 µg/ml puromycin. Protein expression of ADI_AI_ transgene was assessed by Western blot on cell lysates as described before [69] using an anti-HA tag antibody (rat mab clone 3F10; Merck) or anti-ADI alpaca antiserum (see above). As loading control an anti-alpha-tubulin antibody (mouse mab clone DM1A; Merck) was used. Peroxidase conjugated goat anti-mouse, anti-llama or anti-rat antibodies (1:5000, Bethyl laboratories) were used for detection (see above).

To create the CRISPR-based ADI knockout, gRNAs were designed to target position 870 of the ADI gene (GL50803_112103) for insertion of antibiotic resistance cassettes. The gRNA (20 nucleotides) was designed to target the non-template strand (with 4-base overhangs complementary to the vector sequence overhangs using the Benchling software (https://benchling.com/crispr) with a NGG PAM sequence and the *G. duodenalis* ATCC 50803 genome (GenBank Assembly: GCA_000002435.1). gRNA oligonucleotides were annealed and cloned into Bbsl-digested Cas9U6g1pac as previously described [70]. Linear repair templates were designed with a left homology arm (750 bp upstream of the gRNA870 site), an antibiotic resistance cassette, and a right homology arm (750 bp downstream of the gRNA870 site). Two repair templates were designed: one with a blasticidin (bsd) resistance cassette and one with a hygromycin B (hyg) resistance cassette [42,71]. Linear repair templates were synthesized by Twist Bioscience.

The CRISPR plasmid (Cas9U6gipac_112103g870) was electroporated into WBC6 trophozoites (20 μg DNA) as previously described [72]. The Cas9_112103g870 strain was maintained with antibiotic selection (50 μg/ml puromycin). The bsd linear repair template (20 µg) was first electroporated into the Cas9_112103g870 strain. Selection was maintained for the Cas9_112103g870 plasmid with 50 μg/ml puromycin and selection for integration of the repair template started at 75 μg/ml blasticidin, which was increased to 150 μg/ml blasticidin as the electroporated cells recovered. Blasticidin repair template integration was confirmed by PCR and long read sequencing. Next, the hyg linear repair template (20 µg) was electroporated into the Cas9_112103g870 strain that had the bsd repair template integrated at least once in the *Giardia* genome. Selection was maintained for the Cas9 plasmid with 50 μg/ml puromycin and selection for integration of the repair template started at 600 μg/ml hygromycin B, which was increased to 1200 μg/ml hygromycin B as the electroporated cells recovered. The hygromycin B repair template integration was confirmed by PCR and long read sequencing.

The KO strain confirmed to have both the bsd resistance cassette and hyg resistance cassette integrated into the genome with no wild type alleles was cloned by dilution to extinction. The contents of positive wells (those with growth) were transferred first to 6 ml TYI-S-33 medium, and then up to 12 ml TYI-S-33 medium. Clones were screened for complete knockout of the ADI gene by PCR (S13 Fig) using primers that bound to the 5’- and 3’-ends of the ADI gene (112103-F, 5’-AGATAGTCGTCGTGCACCTC -3’; and 112103-R, 5’-CAGATGTCAGCCTGCTCCAG-3‘). Upon insertion of an antibiotic cassette into the ADI gene, the PCR product would be increased by a known amount. One of the clones was selected for downstream analysis.

Complementation of ADI-KO was done by add-back of ADI_AI_ and ADI_AII_, respectively, using the same method as described above for the expression of the additional ADI gene. For this, puromycin resistance gene cassette was first replaced by neomycin resistance gene cassette to generate a pNEO backbone vector (from parental pPAC-ORF16653-HA). Then, the ORF16653-HA sequence was replaced by the ADI variants to generate pNEO-ADI_AI_-3HA or pNEO-ADI_AII_-3HA constructs. These constructs were linearized and transfected into the ADI-KO clone A12 as described above. Selection of transfectants was done using 150 µg/ml neomycin. Complemented strains were confirmed by various PCR approaches (S13 Fig), sequencing of the integrated ADI gene and by Western blot analysis using anti-HA and anti-ADI antibodies as described above.

### ADI structure

For ADI of assemblage AI and assemblage AII isolates, the respective files from AlphaFold2 structure prediction [36] were downloaded and analyzed and illustrated using ChimeraX [73]. Also, Chai-1 [37] was used to predict arginine binding in the active center and to model structural changes by depicted amino acid mutations. The following datasets were used: AF-E2RU36-F1-model_v4 (WB6, assemblage AI) and AF-V6THM7-F1-model_v4 (DH, assemblage AII).

### Descriptive statistics

Data are given as mean ± standard deviations (SDs). Statistical significance was assessed by using paired (two-tailed) *t* test or one-way ANOVA with adequate post hoc tests, if not indicated otherwise. All analyses were performed with GraphPad Prism 9 software (GraphPad Software, Inc.) with a statistical significance level of *P* < 0.05.

### Data depository

ADI sequences presented are available at Genbank under following accession number: PV067229 - PV067249.

## Supporting information

S1 Fig ADI enzyme kinetics of cell extracts from different *Giardia duodenalis* isolates. Of each isolate from Fig 3B three exemplary independent enzymatic assays are shown. Each assay was performed in technical triplicates.
(TIF)

S2 Fig ADI enzyme kinetics of recombinant ADI proteins from different *Giardia duodenalis* assemblages. Three independent enzymatic assays of depicted ADI variants from Fig 3D are shown. Each assay was performed in technical triplicates.
(TIF)

S3 Fig Allelic sequences of ADI of 14 different patient isolates of *G. duodenalis* assemblage AII. Representation of allelic sequences of ADI of 14 different patient isolates of *G. duodenalis* assemblage AII from an internal *G. duodenalis* biobank (Isolates: P029, P034, P064, P157, P168, P203, P212, P316, P324, P361, P368, P392, P407, P644). Sequences were retrieved by PCR and Sanger sequencing and aligned to reference ADI sequence “ADI_WB” of WB isolate (assemblage AI, gene ID “GL50803_112103”). Sequence of “P407” represents an identical sequence found in 11 different AII isolates. This sequence was also identical to the reference sequence of DH isolate (gene ID DHA2_112103). Three patient isolates had one or two additional mutations but at different sites in the sequences.
(TIF)

S4 Fig Allelic sequences of ADI of 15 different patient isolates of *G. duodenalis* assemblage B. Representation of allelic sequences of ADI of 15 different patient isolates of *G. duodenalis* assemblage B from an internal *G. duodenalis* biobank (Isolates: P132, P289, P344, P387, P413, P424, P427, P439, P448, P458, P486, P514, P621, P678, P786). PCR fragments of ADI of each isolate were cloned into pJet vector and transformed into *E. coli*. Plasmids of 10 single clones each were analyzed by Sanger sequencing and ADI sequences aligned to reference ADI sequence “ADI_GS” of GS isolate (assemblage B, gene ID “GL50581_1575”). Due to possible introduction of sequence chimeras during PCR, alleles were only defined “true” for sequences with two or more identical copies within the 10 analyzed clones. This revealed 17 different ADI alleles within the 15 isolates.
(TIF)

S5 Fig ADI protein variants derived from patient isolates of assemblage AII and B. Representation of all ADI protein variants derived from patient isolates of assemblage AII (see S3 Fig) and assemblage B (see S4 Fig) showing the conserved SNPs of assemblage AII sequences at position G167, V449 and L494. For comparison, reference sequences of assemblage AI (WB isolate) and assemblage B (GS isolate) were included. Note, AII_allele_1–4 represent corresponding sequences of P407, P368, P168 and P157, respectively, shown in S3 Fig.
(TIF)

S6 Fig ADI enzyme kinetics of mutagenized recombinant ADI_AI_ protein variants. Three independent enzymatic assays of depicted ADI variants from Fig 4A are shown. Each assay was performed in technical triplicates.
(TIF)

S7 Fig Structural representation of ADI of assemblage AI, including arginine positioned in the active center. Structural representation of ADI_AI_ (Ser167, Ile449, Pro494; wt) and overlay with mutated ADI functionally relevant in the assemblage AII ADI (Gly167, Leu494; 2x mut). Arginine substrate is positioned in the active center as predicted by the algorithm used in Chai-1 [37]. Color code is separated as indicated in the lower picture, RMSD (root mean square deviation) indicates the estimated difference in distance between the two modeled structures. pLDDT (predicted local distance difference test) represents reliability of the prediction.
(TIF)

S8 Fig Reduced encystation efficiency phenotype in P407 (AII) isolate in comparison to WB (AI) isolate is not due to delayed encystation. Encystation efficiency of assemblage AI (WB) and assemblage AII (P407) was compared at 24- and 48-hours post induction of encystation. Three independent experiments are shown, each performed with six replicates. For statistics we used one-way ANOVA and Tukey post hoc test (exact p values are shown).
(TIF)

S9 Fig Determination of arginine concentration in *Giardia* encystation media. To estimate the arginine concentration in *Giardia* encystation media, we added different amounts (0-16 µg) of recombinant ADI_AI_ to 1 ml of encystation medium (EM) and incubated for 24 hours at 37°C and determined the citrulline quantity (which is equimolar to the converted arginine). Normal *Giardia* culture medium (CM, 10 ml batch) was included for comparison. As reference, 1ml commercial RPMI medium with known arginine concentration of 1150 µM was included as well. Shown is one experiment in triplicates.
(TIF)

S10 Fig Assessment of ADI expression in transgenic *G. duodenalis* P407 (assemblage AII) and WB (assemblage AI) strains expressing an additional version of HA-tagged ADI_AI_. Western blot analysis was performed using a custom-made anti-ADI antibody to detect total ADI expression, including endogenous and recombinant HA-tagged ADI_AI_ protein, in cell lysates of depicted transgenic and parental *G. duodenalis* strains. See Fig 5C for Western blot showing detection of HA-tagged ADI_AI_ transgene by an anti-HA antibody. A pan alpha-tubulin antibody was used as a loading control.
(TIF)

S11 Fig Lower cyst excretion of *G. duodenalis* infected mice fed with arginine-free diet. (A) C57BL/6 mice infected with *Giardia duodenalis* assemblage B (GS/H7 strain, ATCC 50581) and fed with a defined arginine-free diet (n = 14) showed significantly lower cyst excretion in the feces (pooled cyst numbers in feces collected day 4–7 post infection) than control animals (n = 13) fed with the defined arginine-repleted diet containing 1% arginine. Note, that the defined diet is less rich and complex than “normal” diet, leading to overall lower cyst excretion. For comparison, mice fed with normal diet following the same infection protocol lead to significintly higher overall cyst excretion per gram feces at day 7 post infection (5.1 ± 1.3 x 10^5^, n = 22). (B) No differences were detected analyzing the genome equivalents by qPCR in tissue of the upper small intestine of the same mice as in (A). For statistics we used Kruskal-Wallis rank sum test.
(TIF)

S12 Fig Depletion of ADI enzymatic function in ADI-KO parasites. ADI enzymatic activity was not detectable (n.d.) in lysates of both ADI-KO clones (E10 and A12) while both WB control strains (“RKI” and “UCD”) showed similar *K*_m_ values. Mean ± SD *K*_m_ values from three independent experiments in triplicates are given. Exemplary enzymatic curves of one experiment are shown on the right site.
(TIF)

S13 Fig PCR approaches confirming ADI-KO mutant and respective ADI_AI_ and ADI_AII_ add-back complemented strains. Schemes illustrate primer positions for PCR approaches and table represents oligo nucleotide sequences. PCR#1 and #2 confirm genomic integration of ADI_AI/AII_ add-back constructs into the TPI (triosephosphate isolarase) genetic locus. The presence of correct ADI-AI and ADI-AII sequence was confirmed by PCR and sequencing approach used to analyze ADI alleles from different *G. duodenalis* isolates (see Methods and S3 Fig). PCR#3: only amplifies original vector construcs with plasmid backbone. PCR#4: confirms presence of the genome integration of constructs containing NEO resistance cassettes. In the ADI-KO and parental WB strain some unspecific PCR products were detected, which do not represent the correct amplicon size of 850 bp. PCR#5: verifys ADI-KO mutant by amplifying ADI gene fragment with primers flanking the antibiotics integration sites. An ADI fragment was amplified that give rise to a size of ~1600 bp in the wildtype (WT, parental WB6 isolate), and of ~2450 bp (blasticidin resistance) and ~3075 bp (hygromycin resistance) in the ADI-KO mutant. Expectedly, in the complemented strains, the wildtype fragment as well as the two ADI-KO fragments are amplified. The latter two are weaker, but clearly visible. Control PCRs without DNA are indicated by H_2_O. PCR products were analyzed on a 1% agarose gel and vizualized using GelGreen reagent (Biotium, Fremont, CA) on a conventional documentation system (Vilbert Fusion FX6).
(TIF)

S1 Table Catalytic efficiency of recombinant ADI variants. Enzymatic parameter (*K*_m_, V_max_) were retrieved from experiments shown in S2 and S6 Figs to calculate k_cat_ and catalytic efficiency k_cat_/K_m._ Statistical tests (for k_cat_/*K*_m_ values) were performed as indicated by using one-way ANOVA and Tukey post hoc test (exact p values are shown).
(PDF)

S1 Data Values used to build graphs. (XLSX)
