# Supplementary figures and images for: Arginine metabolism has a pivotal function for the encystation of Giardia duodenalis
Source: PLoS Pathog. 2026 Jan 8;22(1):e1013851. doi: 10.1371/journal.ppat.1013851 (PMC12810918; doi:10.1371/journal.ppat.1013851)

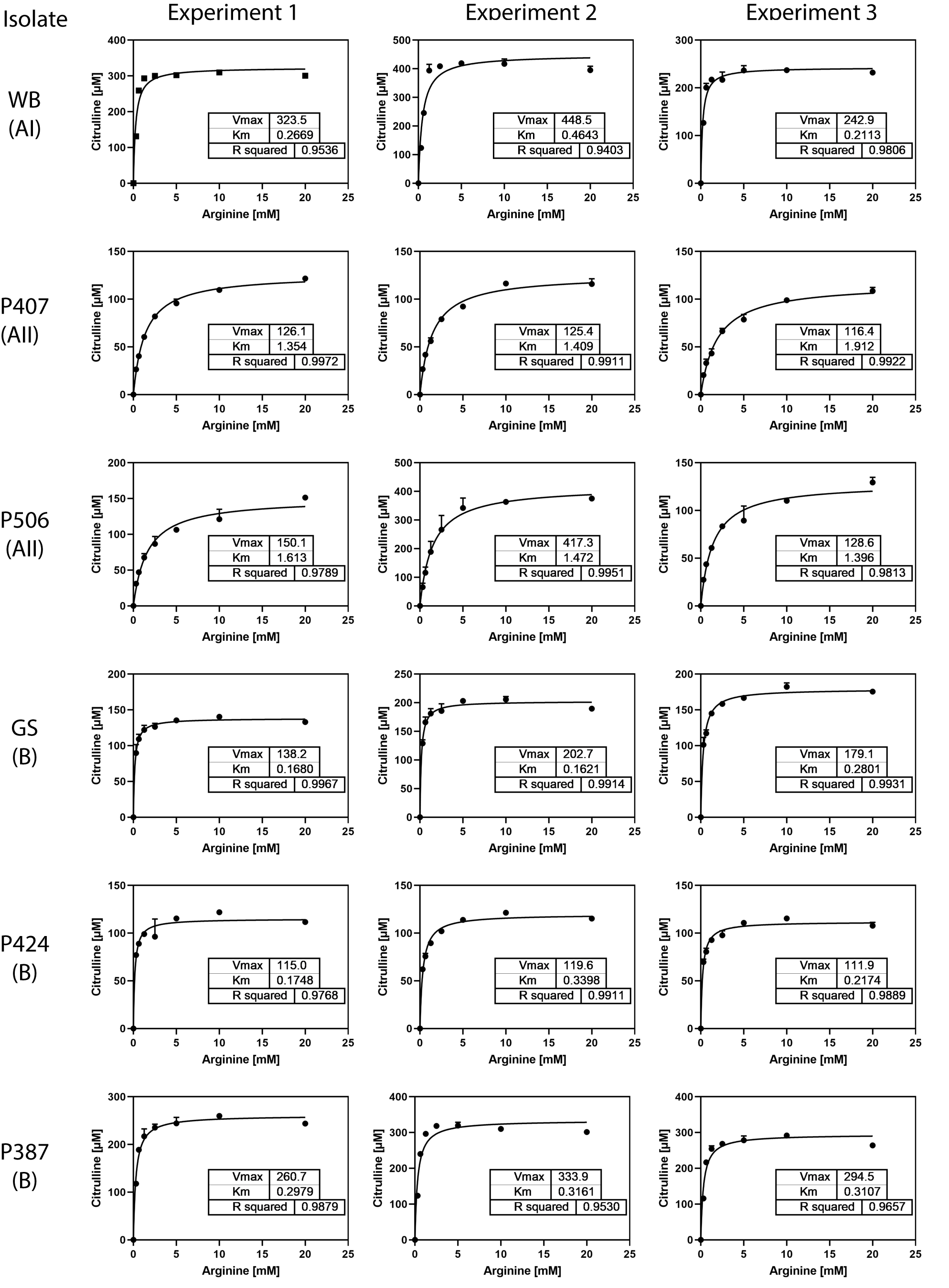

Supplement: S1 Fig — Of each isolate from Fig 3B three exemplary independent enzymatic assays are shown. Each assay was performed in technical triplicates. (TIF) [file ppat.1013851.s001.tif]

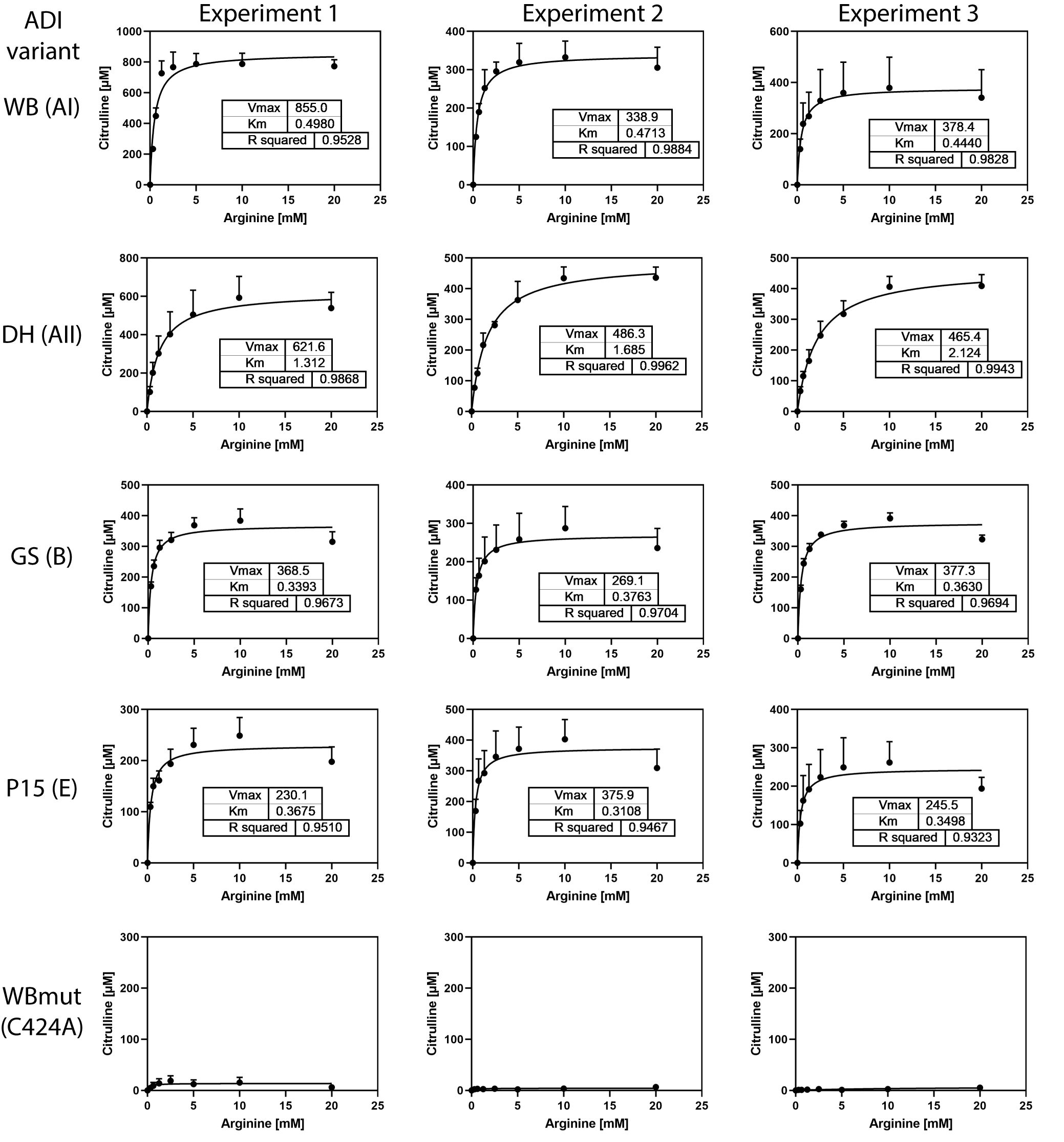

Supplement: S2 Fig — Three independent enzymatic assays of depicted ADI variants from Fig 3D are shown. Each assay was performed in technical triplicates. (TIF) [file ppat.1013851.s002.tif]

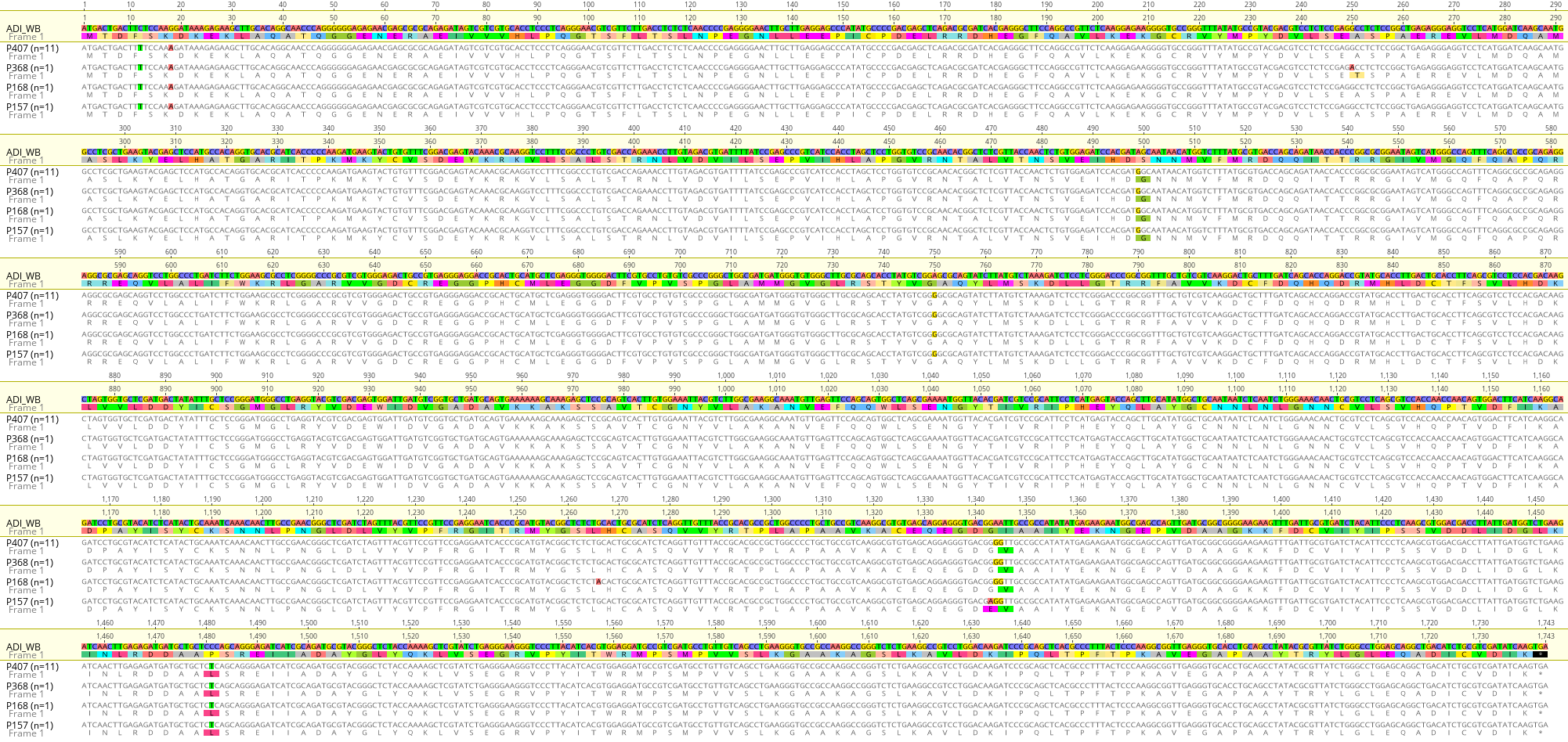

Supplement: S3 Fig — Representation of allelic sequences of ADI of 14 different patient isolates of G. duodenalis assemblage AII from an internal G. duodenalis biobank (Isolates: P029, P034, P064, P157, P168, P203, P212, P316, P324, P361, P368, P392, P407, P644). Sequences were retrieved by PCR and Sanger sequencing and aligned to reference ADI sequence “ADI_WB” of WB isolate (assemblage AI, gene ID “GL50803_112103”). Sequence of “P407” represents an identical sequence found in 11 different AII isolates. This sequence was also identical to the reference sequence of DH isolate (gene ID DHA2_112103). Three patient isolates had one or two additional mutations but at different sites in the sequences. (TIF) [file ppat.1013851.s003.tif]

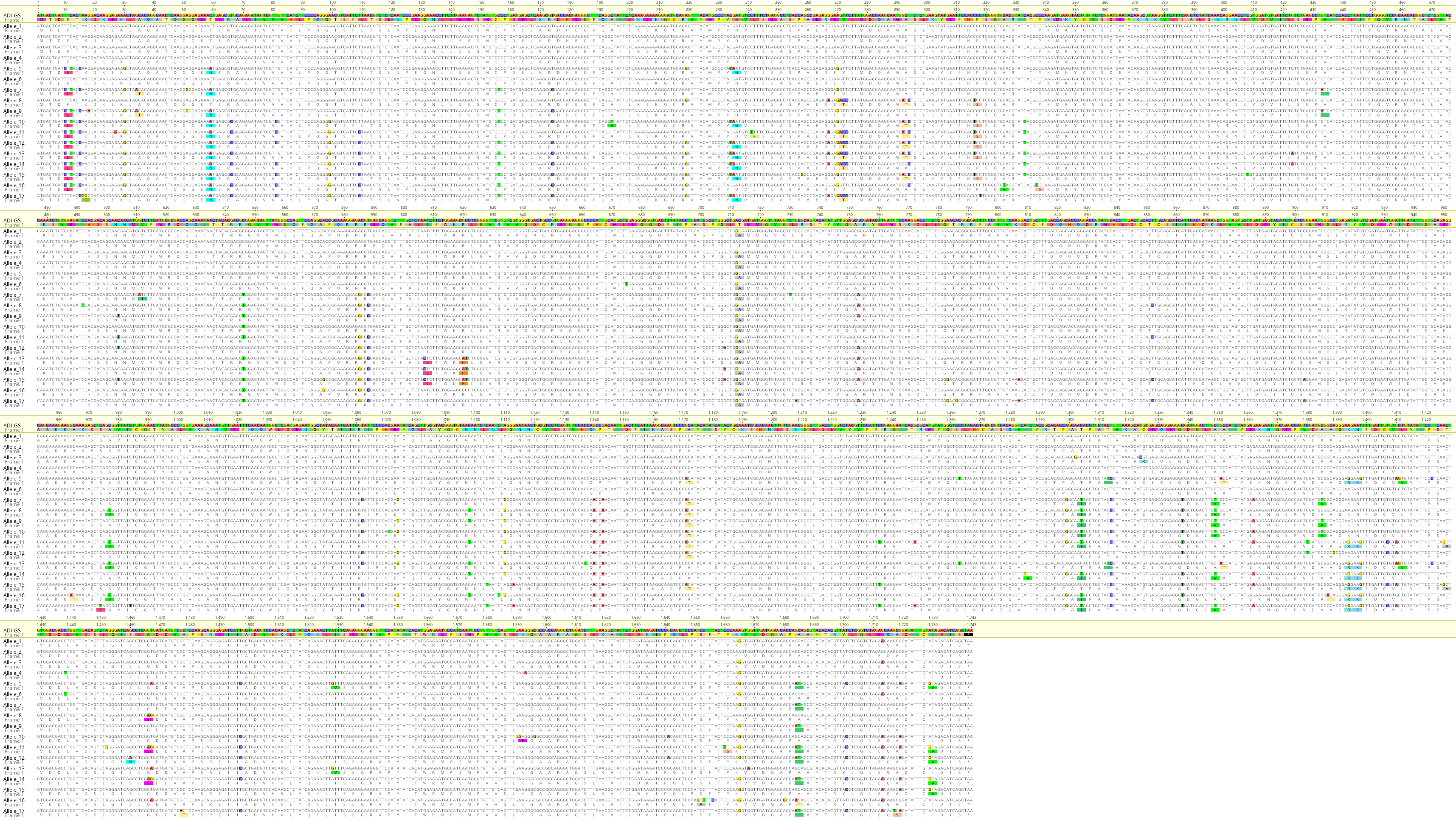

Supplement: S4 Fig — Representation of allelic sequences of ADI of 15 different patient isolates of G. duodenalis assemblage B from an internal G. duodenalis biobank (Isolates: P132, P289, P344, P387, P413, P424, P427, P439, P448, P458, P486, P514, P621, P678, P786). PCR fragments of ADI of each isolate were cloned into pJet vector and transformed into E. coli. Plasmids of 10 single clones each were analyzed by Sanger sequencing and ADI sequences aligned to reference ADI sequence “ADI_GS” of GS isolate (assemblage B, gene ID “GL50581_1575”). Due to possible introduction of sequence chimeras during PCR, alleles were only defined “true” for sequences with two or more identical copies within the 10 analyzed clones. This revealed 17 different ADI alleles within the 15 isolates. (TIF) [file ppat.1013851.s004.tif]

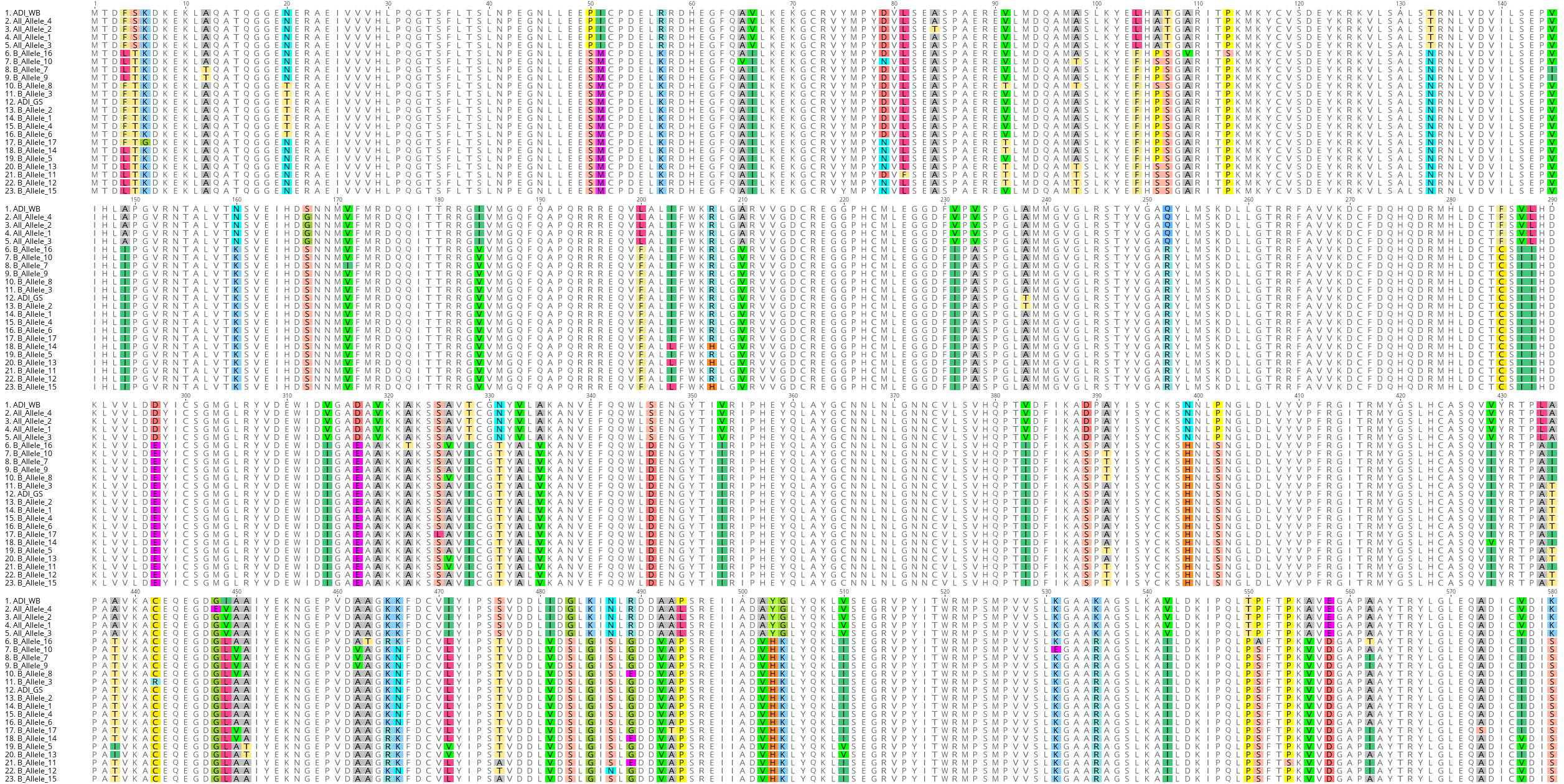

Supplement: S5 Fig — Representation of all ADI protein variants derived from patient isolates of assemblage AII (see S3 Fig) and assemblage B (see S4 Fig) showing the conserved SNPs of assemblage AII sequences at position G167, V449 and L494. For comparison, reference sequences of assemblage AI (WB isolate) and assemblage B (GS isolate) were included. Note, AII_allele_1–4 represent corresponding sequences of P407, P368, P168 and P157, respectively, shown in S3 Fig. (TIF) [file ppat.1013851.s005.tif]

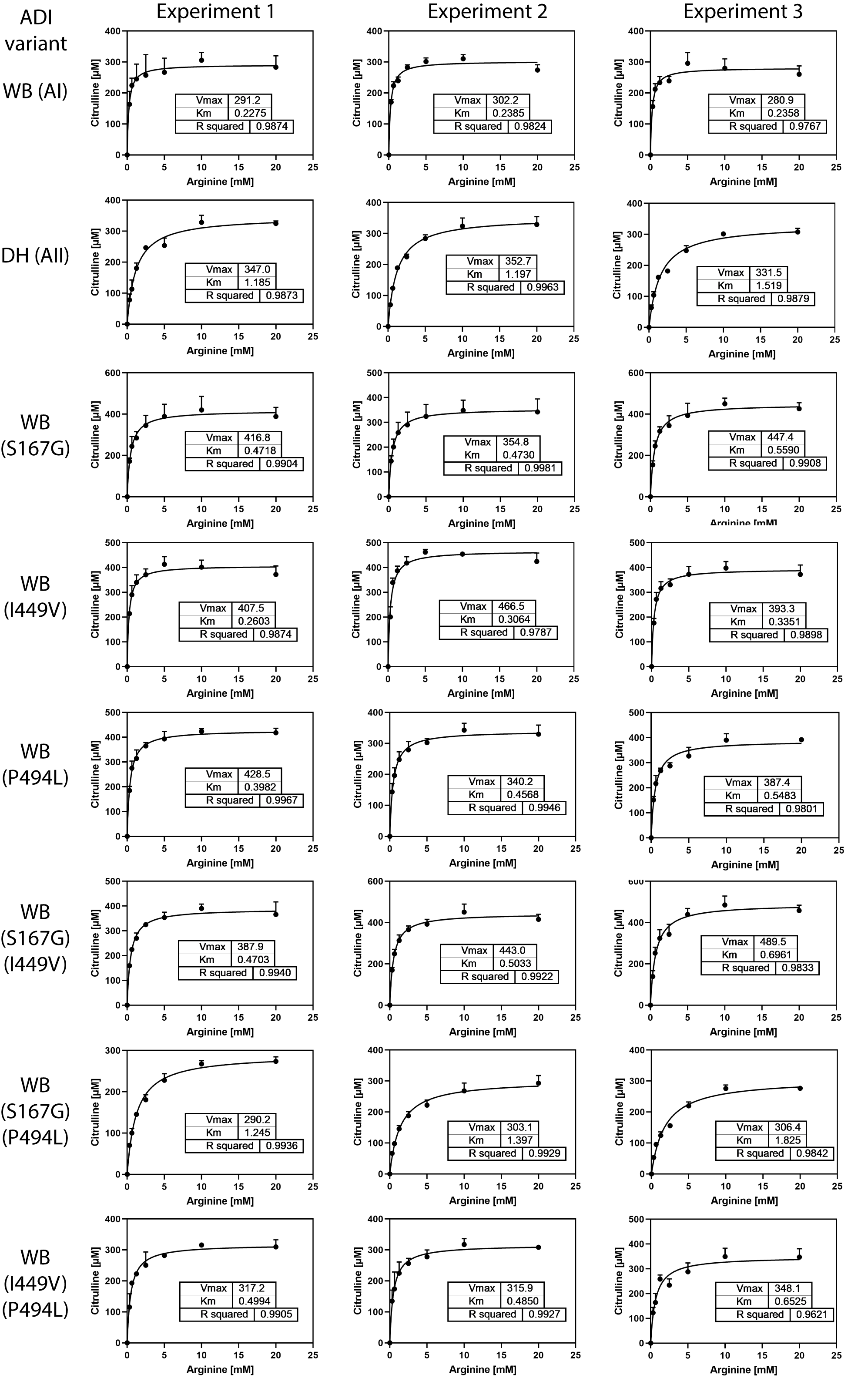

Supplement: S6 Fig — Three independent enzymatic assays of depicted ADI variants from Fig 4A are shown. Each assay was performed in technical triplicates. (TIF) [file ppat.1013851.s006.tif]

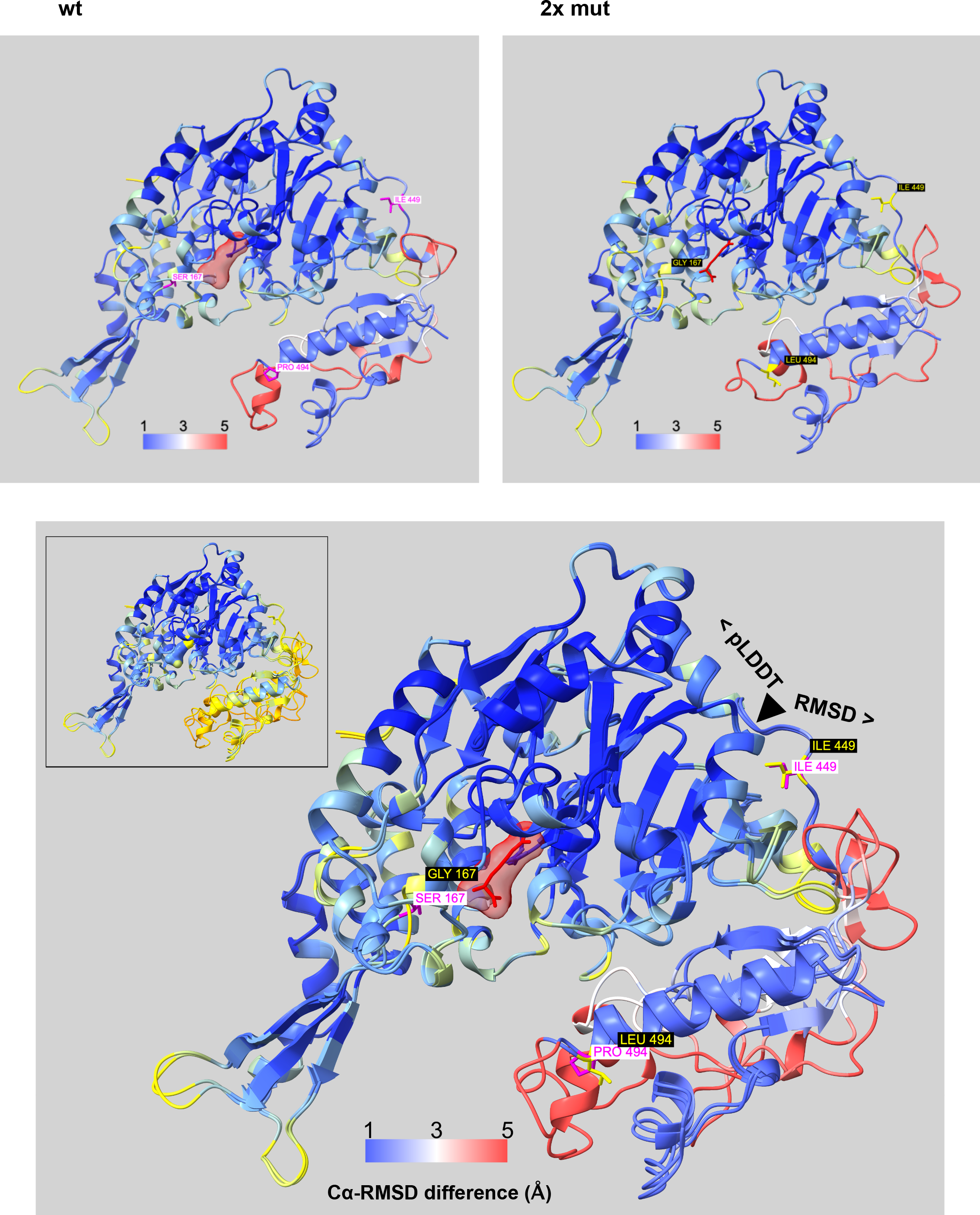

Supplement: S7 Fig — Structural representation of ADIAI (Ser167, Ile449, Pro494; wt) and overlay with mutated ADI functionally relevant in the assemblage AII ADI (Gly167, Leu494; 2x mut). Arginine substrate is positioned in the active center as predicted by the algorithm used in Chai-1 [37]. Color code is separated as indicated in the lower picture, RMSD (root mean square deviation) indicates the estimated difference in distance between the two modeled structures. pLDDT (predicted local distance difference test) represents reliability of the prediction. (TIF) [file ppat.1013851.s007.tif]

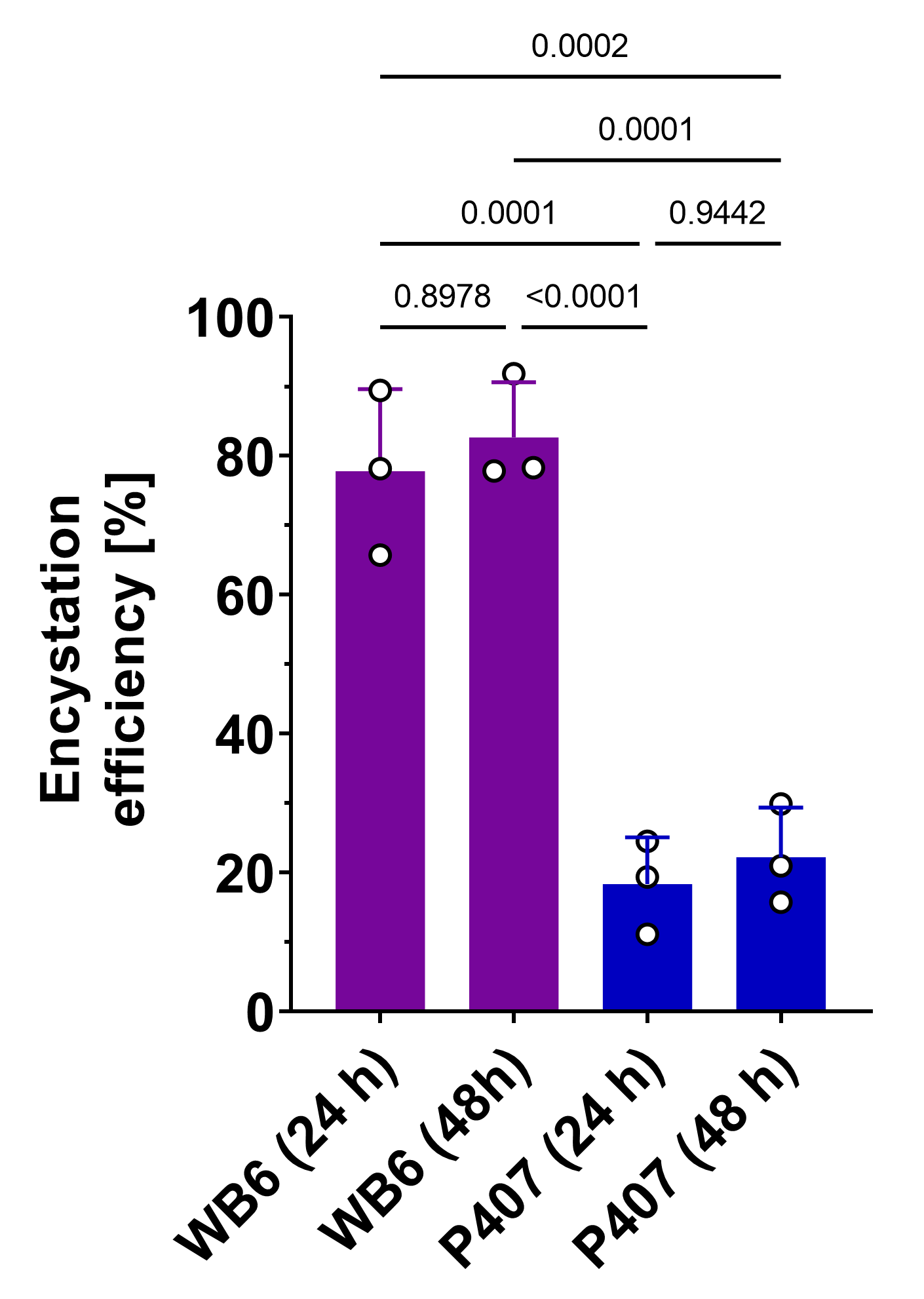

Supplement: S8 Fig — Encystation efficiency of assemblage AI (WB) and assemblage AII (P407) was compared at 24- and 48-hours post induction of encystation. Three independent experiments are shown, each performed with six replicates. For statistics we used one-way ANOVA and Tukey post hoc test (exact p values are shown). (TIF) [file ppat.1013851.s008.tif]

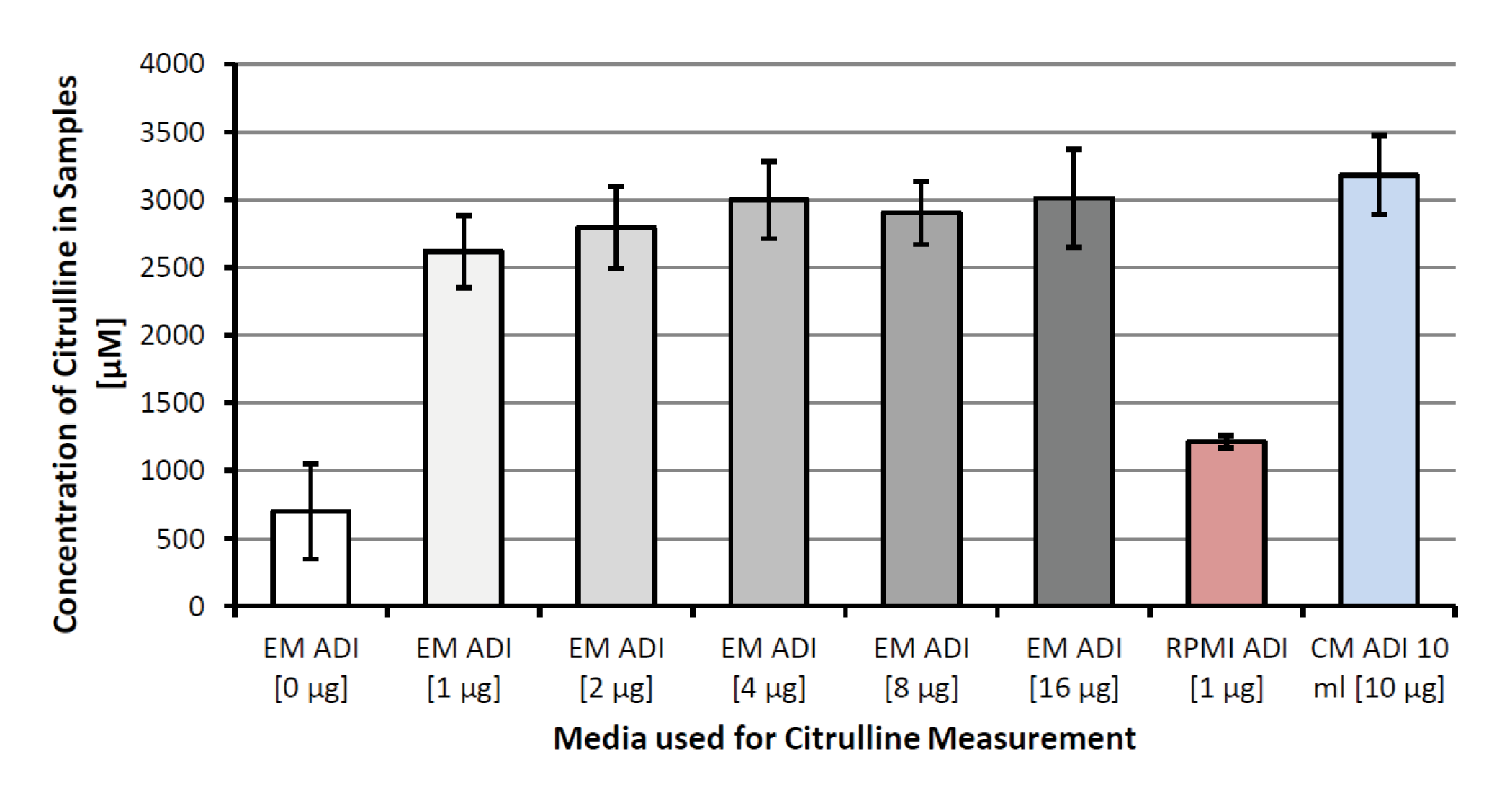

Supplement: S9 Fig — To estimate the arginine concentration in Giardia encystation media, we added different amounts (0-16 µg) of recombinant ADIAI to 1 ml of encystation medium (EM) and incubated for 24 hours at 37°C and determined the citrulline quantity (which is equimolar to the converted arginine). Normal Giardia culture medium (CM, 10 ml batch) was included for comparison. As reference, 1ml commercial RPMI medium with known arginine concentration of 1150 µM was included as well. Shown is one experiment in triplicates. (TIF) [file ppat.1013851.s009.tif]

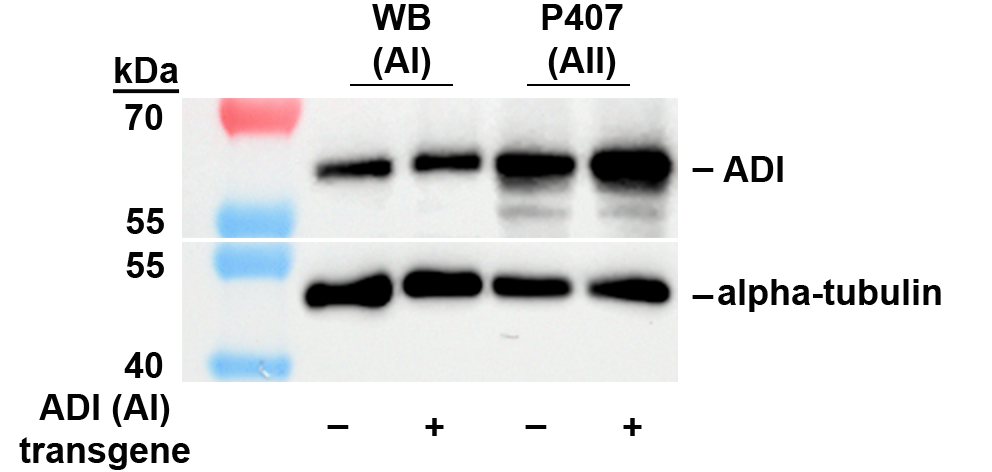

Supplement: S10 Fig — Western blot analysis was performed using a custom-made anti-ADI antibody to detect total ADI expression, including endogenous and recombinant HA-tagged ADIAI protein, in cell lysates of depicted transgenic and parental G. duodenalis strains. See Fig 5C for Western blot showing detection of HA-tagged ADIAI transgene by an anti-HA antibody. A pan alpha-tubulin antibody was used as a loading control. (TIF) [file ppat.1013851.s010.tif]

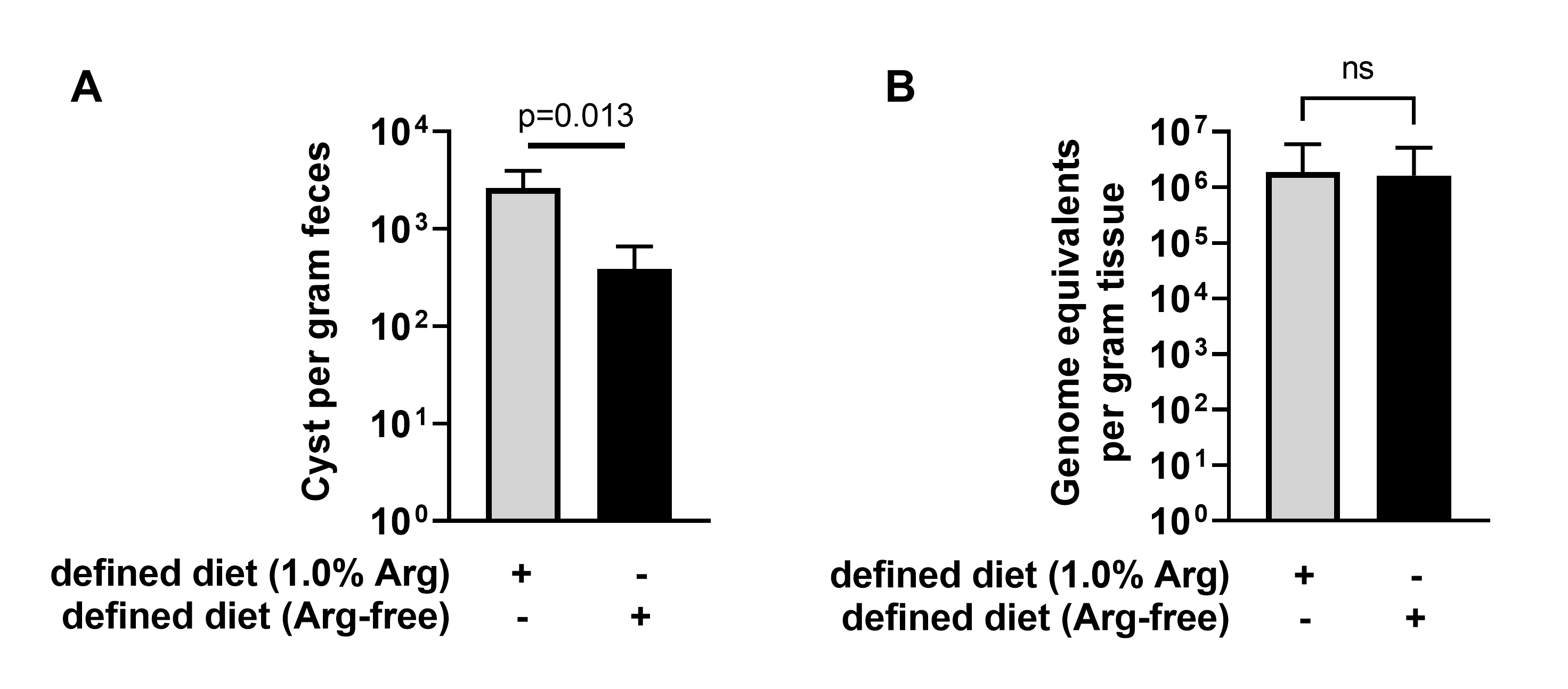

Supplement: S11 Fig — (A) C57BL/6 mice infected with Giardia duodenalis assemblage B (GS/H7 strain, ATCC 50581) and fed with a defined arginine-free diet (n = 14) showed significantly lower cyst excretion in the feces (pooled cyst numbers in feces collected day 4–7 post infection) than control animals (n = 13) fed with the defined arginine-repleted diet containing 1% arginine. Note, that the defined diet is less rich and complex than “normal” diet, leading to overall lower cyst excretion. For comparison, mice fed with normal diet following the same infection protocol lead to significintly higher overall cyst excretion per gram feces at day 7 post infection (5.1 ± 1.3 x 10^5^, n = 22). (B) No differences were detected analyzing the genome equivalents by qPCR in tissue of the upper small intestine of the same mice as in (A). For statistics we used Kruskal-Wallis rank sum test. (TIF) [file ppat.1013851.s011.tif]

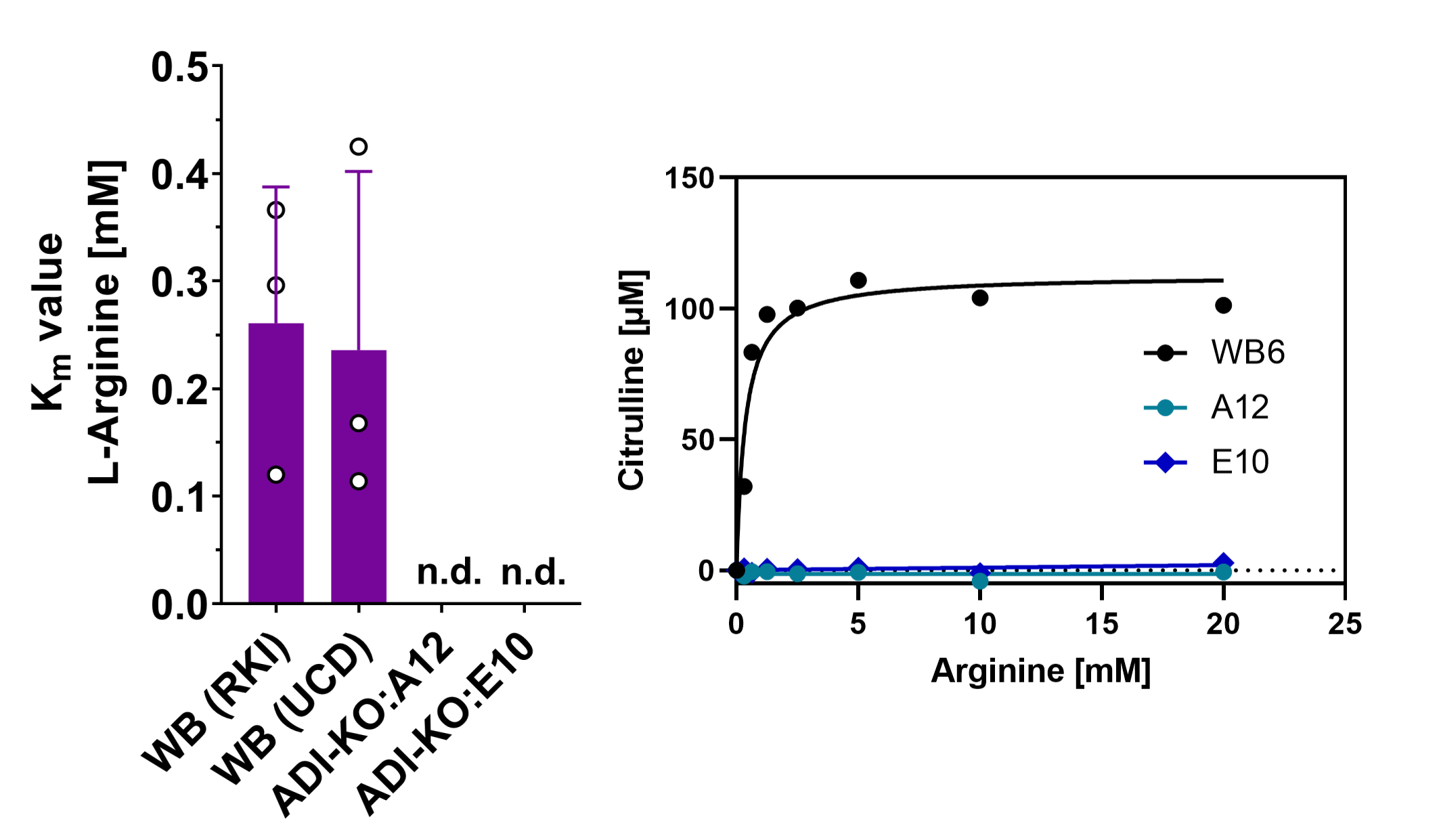

Supplement: S12 Fig — ADI enzymatic activity was not detectable (n.d.) in lysates of both ADI-KO clones (E10 and A12) while both WB control strains (“RKI” and “UCD”) showed similar Km values. Mean ± SD Km values from three independent experiments in triplicates are given. Exemplary enzymatic curves of one experiment are shown on the right site. (TIF) [file ppat.1013851.s012.tif]

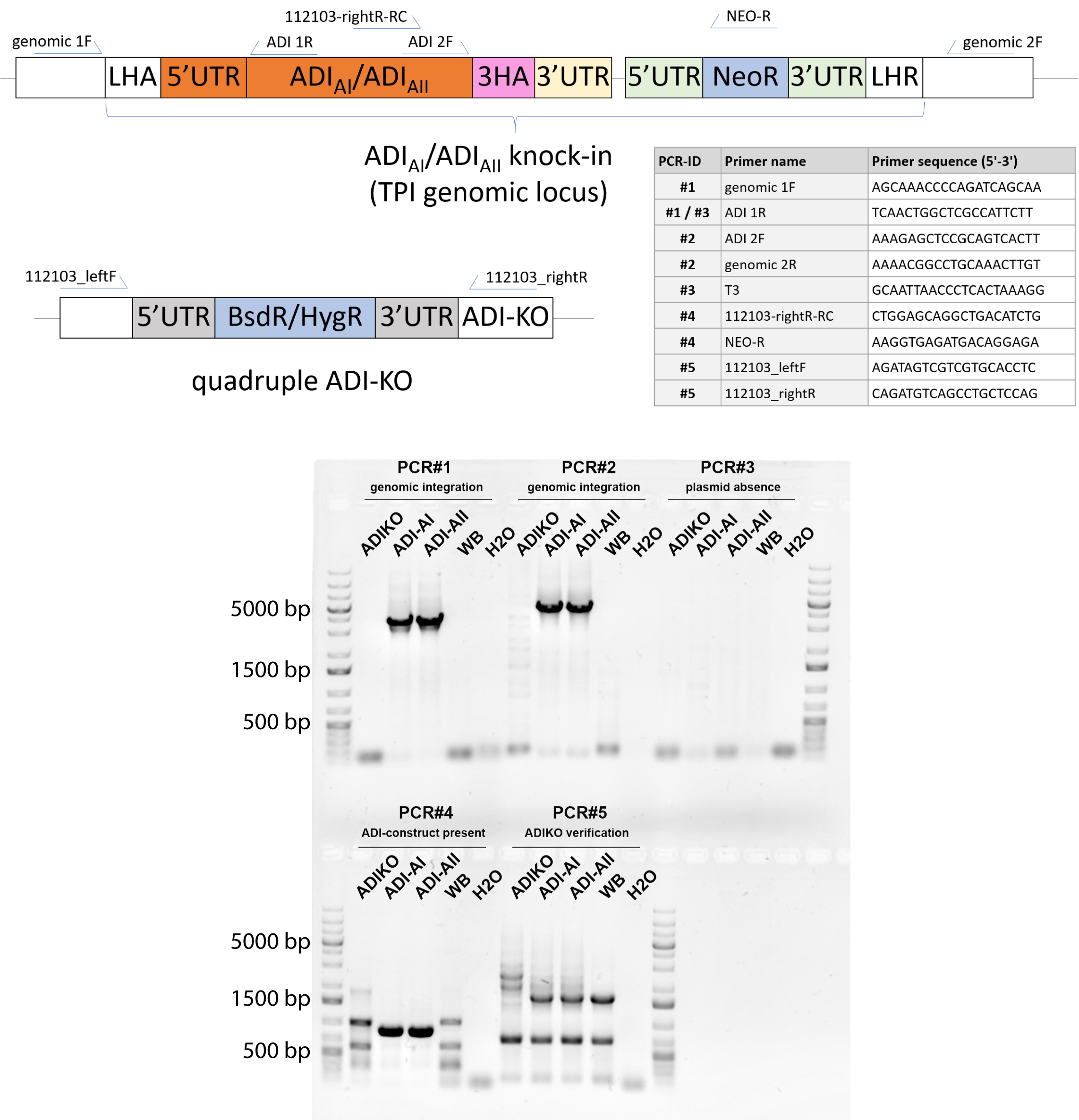

Supplement: S13 Fig — Schemes illustrate primer positions for PCR approaches and table represents oligo nucleotide sequences. PCR#1 and #2 confirm genomic integration of ADIAI/AII add-back constructs into the TPI (triosephosphate isolarase) genetic locus. The presence of correct ADI-AI and ADI-AII sequence was confirmed by PCR and sequencing approach used to analyze ADI alleles from different G. duodenalis isolates (see Methods and S3 Fig). PCR#3: only amplifies original vector construcs with plasmid backbone. PCR#4: confirms presence of the genome integration of constructs containing NEO resistance cassettes. In the ADI-KO and parental WB strain some unspecific PCR products were detected, which do not represent the correct amplicon size of 850 bp. PCR#5: verifys ADI-KO mutant by amplifying ADI gene fragment with primers flanking the antibiotics integration sites. An ADI fragment was amplified that give rise to a size of ~1600 bp in the wildtype (WT, parental WB6 isolate), and of ~2450 bp (blasticidin resistance) and ~3075 bp (hygromycin resistance) in the ADI-KO mutant. Expectedly, in the complemented strains, the wildtype fragment as well as the two ADI-KO fragments are amplified. The latter two are weaker, but clearly visible. Control PCRs without DNA are indicated by H2O. PCR products were analyzed on a 1% agarose gel and vizualized using GelGreen reagent (Biotium, Fremont, CA) on a conventional documentation system (Vilbert Fusion FX6). (TIF) [file ppat.1013851.s013.tif]
