# Supplementary material for: Arginine metabolism has a pivotal function for the encystation of Giardia duodenalis
Source: PLoS Pathog. 2026 Jan 8;22(1):e1013851. doi: 10.1371/journal.ppat.1013851 (PMC12810918; doi:10.1371/journal.ppat.1013851)
Supplement: S1 Table — Enzymatic parameter (Km, Vmax) were retrieved from experiments shown in S2 and S6 Figs to calculate kcat and catalytic efficiency kcat/Km. Statistical tests (for kcat/Km values) were performed as indicated by using one-way ANOVA and Tukey post hoc test (exact p values are shown). (PDF) [file ppat.1013851.s014.pdf]

Catalytic efficiency of recombinant ADI variants.

**ADI variant**	**k_{cat} (s^{-1})**	**K_m (mM)**	**k_{cat}/K_m (M^{-1} s^{-1})**	**p value (k_{cat}/K_m)**		
				vs. AI	vs. AII	vs. B
Recombinant ADI variants of different assemblages (see Fig 3D; S2 Fig)						
WB (AI)	5.57 ± 2.02	0.47 ± 0.03	$1.17 \pm 0.37 \times 10^4$			
DH (AII)	2.95 ± 0.43	1.71 ± 0.41	$0.18 \pm 0.07 \times 10^4$	0,0013		
GS (B)	1.99 ± 0.16	0.36 ± 0.02	$0.55 \pm 0.07 \times 10^4$	0,0217	0,1814	
P15 (E)	1.27 ± 0.30	0.34 ± 0.03	$0.41 \pm 0.12 \times 10^4$	0,0066	0,5500	0,7984
WB_{mut} (C424A)	-	-	-			
Mutagenized recombinant ADI variants of WB (AI) (see Fig 4A; S6 Fig)						
WB (AI)	2.92 ± 0.11	0.23 ± 0.01	$1.25 \pm 0.05 \times 10^4$		<0,0001	
DH (AII)	2.43 ± 0.08	1.30 ± 0.19	$0.19 \pm 0.03 \times 10^4$			
WB (S167G)	4.10 ± 0.47	0.50 ± 0.05	$0.82 \pm 0.07 \times 10^4$		<0,0001	
WB (I449V)	3.86 ± 0.36	0.30 ± 0.04	$1.30 \pm 0.20 \times 10^4$		<0,0001	
WB (P494L)	3.51 ± 0.40	0.47 ± 0.08	$0.77 \pm 0.18 \times 10^4$		<0,0001	
WB (S167G, I449V)	4.14 ± 0.48	0.56 ± 0.12	$0.75 \pm 0.08 \times 10^4$		<0,0001	
WB (S167G, P494L)	2.24 ± 0.06	1.49 ± 0.30	$0.15 \pm 0.03 \times 10^4$		0,9976	
WB (I449V, P494L)	3.13 ± 0.18	0.55 ± 0.09	$0.58 \pm 0.06 \times 10^4$		0,0022	
